# Profiling the venom gland transcriptomes of Costa Rican snakes by 454 pyrosequencing

**DOI:** 10.1186/1471-2164-12-259

**Published:** 2011-05-23

**Authors:** Jordi Durban, Paula Juárez, Yamileth Angulo, Bruno Lomonte, Marietta Flores-Diaz, Alberto Alape-Girón, Mahmood Sasa, Libia Sanz, José M Gutiérrez, Joaquín Dopazo, Ana Conesa, Juan J Calvete

**Affiliations:** 1 Consejo Superior de Investigaciones Científicas, Jaime Roig 11, 46010 Valencia. Spain; 2 Instituto Clodomiro Picado, Universidad de Costa Rica, San José, Costa Rica; 3 Centro de Investigaciones en Estructuras Microscópicas, Universidad de Costa Rica, San José, Costa Rica; 4 Centro de Investigación Príncipe Felipe, Valencia. Spain

**Keywords:** Snake venom gland transcriptomics, next generation high-throughput DNA sequencing, 454 pyrosequencing, bioinformatic analysis, Costa Rican snakes, *Bothrops asper*, Bothriechis, Atropoides, Crotalus, Cerrophidion

## Abstract

**Background:**

A long term research goal of venomics, of applied importance for improving current antivenom therapy, but also for drug discovery, is to understand the pharmacological potential of venoms. Individually or combined, proteomic and transcriptomic studies have demonstrated their feasibility to explore in depth the molecular diversity of venoms. In the absence of genome sequence, transcriptomes represent also valuable searchable databases for proteomic projects.

**Results:**

The venom gland transcriptomes of 8 Costa Rican taxa from 5 genera (*Crotalus*, *Bothrops*, *Atropoides*, *Cerrophidion*, and *Bothriechis*) of pitvipers were investigated using high-throughput 454 pyrosequencing. 100,394 out of 330,010 masked reads produced significant hits in the available databases. 5.165,220 nucleotides (8.27%) were masked by RepeatMasker, the vast majority of which corresponding to class I (retroelements) and class II (DNA transposons) mobile elements. BLAST hits included 79,991 matches to entries of the taxonomic suborder *Serpentes*, of which 62,433 displayed similarity to documented venom proteins. Strong discrepancies between the transcriptome-computed and the proteome-gathered toxin compositions were obvious at first sight. Although the reasons underlaying this discrepancy are elusive, since no clear trend within or between species is apparent, the data indicate that individual mRNA species may be translationally controlled in a species-dependent manner. The minimum number of genes from each toxin family transcribed into the venom gland transcriptome of each species was calculated from multiple alignments of reads matched to a full-length reference sequence of each toxin family. Reads encoding ORF regions of Kazal-type inhibitor-like proteins were uniquely found in *Bothriechis schlegelii *and *B. lateralis *transcriptomes, suggesting a genus-specific recruitment event during the early-Middle Miocene. A transcriptome-based cladogram supports the large divergence between *A. mexicanus *and *A. picadoi*, and a closer kinship between *A. mexicanus *and *C. godmani*.

**Conclusions:**

Our comparative next-generation sequencing (NGS) analysis reveals taxon-specific trends governing the formulation of the venom arsenal. Knowledge of the venom proteome provides hints on the translation efficiency of toxin-coding transcripts, contributing thereby to a more accurate interpretation of the transcriptome. The application of NGS to the analysis of snake venom transcriptomes, may represent the tool for opening the door to systems venomics.

## Background

Venomous snakes of the families Viperidae and Elapidae possess paired specialized venom glands located in the upper jaw ventral and posterior to the eyes [1] that produce an arsenal of toxins [2,3], which they inject into prey tissues through high-pressure delivery fangs [4]. Within the reptile clade Toxicofera, venom was a single ancient innovation [5]. Snake venom toxins are the result of recruitment events by which ordinary genes were duplicated, and the new genes selectively expressed in the venom gland and amplified to multigene families with extensive neofunctionalization throughout ~100 million years of evolution [5,6]. Given the central role that diet has played in the adaptive radiation of snakes [7], venom thus represents a key adaptation that has played an important role in the diversification of snakes.

Envenoming by snakebites constitutes a highly relevant, though neglected, public health issue on a global basis [8], as there are venomous organisms in every continent and almost every country. However, venomous animals are particularly abundant in tropical regions, the kitchen of evolution. Arthropod stings constitute the most common cause of envenoming by animals, although around 80% of the more than 150.000 yearly deaths by envenomings worldwide are caused by snakebites, followed by scorpion stings, which cause 15% [9,10]. The venoms of extant snakes comprise complex cocktails of proteins tailored by Natural Selection to act on vital systems of the prey [11]. Medical uses of venoms are well documented in folk remedies and in Western and Chinese traditional medicine [12]. However, despite their remarkable potency and high degree of target specificity, only in the last decades have toxins been increasingly used as pharmacological tools, and it has been realized that venoms represent a vast and essentially untapped resource of preoptimized lead molecules for the medicinal chemist [12-17].

Adequate treatment of snakebites is critically dependent on the ability of antivenoms to neutralize the lethal and tissue-damaging toxins, reversing thereby the signs of envenoming [18,19]. A long term research goal of venomics, of applied importance for improving current antivenom therapy, but also for drug discovery, is to understand the molecular mechanisms and evolutionary forces that underlie the enormous pharmacological potential of venoms [12]. Individually or combined, proteomic and transcriptomic studies have demonstrated their feasibility to explore in depth the molecular diversity of venoms [[20-28], and references therein]. In the absence of genome sequence, transcriptomes represent also valuable searchable databases for proteomic projects.

Since the pioneer report by Ho and co-workers in 1995 [29], snake venom transcriptomic studies have relied on sequencing DNA clones randomly picked from a cDNA library constructed by reverse transcription of the RNA molecules expressed in the venom gland [25]. The partial cDNA sequences derived from expressed genes, also known as Expressed Sequence Tags (ESTs) [30], cluster into groups of contiguous sequences (contigs), which eventually cover the entire extension of the original RNA molecule. In addition, the number of ESTs clustered into a contig is proportional to the transcriptional level of the parent RNA in the venom gland [25]. However, in the few instances in which transcriptomics and proteomics databases have been compared [26,27,31,32], a low degree of concordance has been reported. The occurrence of non-venom-secreted toxin transcripts might indicate that these messengers exhibit an individual or a temporal expression pattern over the life time of the snake [33], or may encode very low-abundance venom proteins. On the other hand, the presence in the venom of toxins not represented in the transcriptome clearly indicates that construction of the cDNA library was biased, i.e. due to the necessary fractionating steps to avoid interfering substances like short, partial length 3'-end cDNAs and adapter sequences [34]. A second bias of cDNA libraries is the potential of the mRNA transcript in plasmids to be partially expressed in their bacterial cells with lethal effects [35]. Moreover, smaller cDNA fragments are over represented compared to larger ones, due to the higher transformation efficiency of smaller plasmids [35].

The high demand for low-cost sequencing has driven the development of high-throughput next-generation sequencing (NGS) technologies such as 454 Roche, Illumina's Solexa, and Applied Biosystems' SOLiD, and and most recently released Helicos HeliScope platforms as alternatives to the classical chain-termination Sanger method of DNA sequencing for the qualitative and quantitative analyses of transcriptomes [36,37]. NGS technologies are revolutionizing the field of transcriptomics by rapidly reducing the time and cost per base sequenced [38]. For example, snake venom gland transcriptomes reported are typically arranged from few hundreds to few thousands ESTs [25]. The largest transcriptome database was assembled from 8696 ESTs (mean read length of 398 bp) from *Deinagkistrodon acutus *venom gland [39]. Only very recently Rokyta and colleagues [40] reported a high-throughput venom gland transcriptome of the Eastern Diamond Rattlesnake (*Crotalus adamanteus*) using Roche 454 sequencing technology. 82,621 reads were singletons, and the remaining 552,863 reads were assembled into 24,773 contigs of average length 513 nucleotides [40]. NGS technologies applied to the transcriptomic analysis of non-model species has the advantage of providing a genome-wide, unbiased insight into the transcriptome [41]. However, NGS techniques applied to non-model species, which like snakes lack a suitable reference genome sequence, are not devoid of limitations. NGS technologies provide shorter and more error-prone reads than Sanger sequencing, making transcript assembly a challenging bioinformatic task, which frequently yields a large set of contigs but a fragmented transcriptome [38,41]. Here, we report the application of the 454 platform to infer the venom gland transcriptomes of Costa Rican snakes, *Bothrops asper *(from Caribbean (car) and Pacific (pac) populations), *Bothriechis lateralis*, *Bothriechis schlegelii*, *Atropoides picadoi*, *Atropoides mexicanus*, *Crotalus simus*, and *Cerrophidion godmani*, with special emphasis on the strategy used to assemble and analyze the gathered DNA sequences. Although the average length of singletons (174 bp) and contigs (208.2 bp), and the low coverage of reads per contig (6), prevented the generation of definitive and reliable full-length gene sequences, our results provide a deep comparison of the transcriptional activity of the venom glands of these medically relevant species in Central America [42,43].

## Results and Discussion

### 454 sequencing statistics and annotation of transcripts

The eight venom gland single-strand cDNA libraries were sequenced using a multiplex strategy. To this end, each cDNA library was barcoded with a unique 10-base sequence (MID, Multiplex IDentifier) that is recognized by the sequencing analysis software, allowing for automated sorting of MID-containing reads. A total of 334,540 reads (amounting ~ 62 Mb of 8 snake venom gland transcriptomes) were simultaneously sequenced in two runs of the Genome Sequencer FLX System. Raw sequencing data are archived under accession number SRP003780 in the NCBI Sequence Read Archive (SRA, http://www.ncbi.nlm.nih.gov/Traces/sra) [44]. Accession codes by species are SRS117169.3 (*B. asper *(car)); SRS117211.3 (*B. asper *(pac)); SRS117214.2 (*C. simus*); SRS117215.1 (*A. picadoi*); SRS117216.1 (*A. mexicanus*); SRS117217.1 (*C. godmani*); SRS117218.1 (*B. lateralis*); and SRS117219.2 (*B. schlegelii*). The first run included only cDNA from *B. asper *from the Caribbean versant of Costa Rica, and was performed as a test run. The second run was done using cDNA from all the species investigated. The average length per read was 186.6 bases (max. 645 bp, and only 3.27% of reads < 50 bp), and this figure is in keeping with other 454 transcriptomic reports conducted in non-model species [45]. 4530 reads could not be assigned due to sequencing errors in the 3' 10-mer label. This figure corresponds to a sequencing error rate of 1.35%, which is higher than that (~ 0.04%) reported in other studies^35,44^. In addition, 5,165,220 nucleotides (8.27%) were masked with N's characters by RepeatMasker. The vast majority of sequence elements masked are class I (retroelements) and class II (DNA transposons) mobile elements [46-48]. Retroelements may have a profound impact on the plasticity of the host genomes [49], i.e. modulating transcription of immediately downstream host genes [50,51]. The bulk (64%) of mobile elements identified in the snake venom gland transcriptomes investigated here are retrotransposons (Additional file 1: Table S1). Retrotransposable elements have been previously reported in the transcriptomes of *Bothrops insularis *(4.1% of ESTs) [52], *Lachesis muta *(0.3%) [53], and *Philodryas olfersii *(4.1%) [54], and in PLA2 genes from the venom gland of *Vipera ammodytes *[55,56] and *Protobothrops flavoviridis *[57]. In the context of multigene toxins, which like the snake venom PLA_2_s are evolving under strong positive adaptive selection [58-60], it is worth mentioning that transposable elements are overrepresented in the mRNAs of rapidly evolving genes [61], suggesting that they have played a role in the diversification and expansion of these gene families [61,62].

The 454 sequencing run yielded a total of 330,010 masked reads, which were distributed among the 8 venom gland transcriptomes as displayed in Additional file 1: Table S2. In the absence of any reference genome to guide the assembly, the sets of reads of each species were separately processed with program Newbler, the *de novo *assembler tool of the 454 Sequencing platform. However, only 58.4% of all reads clustered into 31,025 contigs (average length of the contigs was 208.2 bp; average number of reads per contig = 6), of which 43% comprised only 2 reads. The program also returned 103,357 singletons (mean length, 174.4 bp). Employing other assembler programs, such as MIRA (http://www.chevreux.org/projects_mira.html) or Velvet [63], and using different settings (i.e. Velvet: hash-length 21 or 31; MIRA: job normal or accurate), did not improve the assembly performance. The transcriptome assembly problem has been documented [64,65], particularly for organisms without a reference genome database. Because of the low data compression gained in the assembly step and the small difference between contigs and reads mean length, bioinformatic processing of the 454 sequence data was performed on whole sets of unassembled reads. The set of 330,010 reads was searched against non-redundant GenBank databases using BLASTX and BLASTN algorithms to identify similar sequences with an e-value cutoff <10^-3^. 100,394 (30.4%) produced significant hits (Additional file 1: Table S2). The high percentage of reads without significant similarity to any known sequence is in line with previous transcriptomic studies. Hence, the 8696 high quality ESTs from a non-normalized cDNA library of *D. acutus *were assembled into 2855 clusters, of which only 45.60% matched known sequences and 54.40% had no match to any known sequence in Genbank [39,66].

BLAST hits included 79,991 matches to entries of the taxonomic suborder *Serpentes*, of which 62,433 (62% of BLAST hits) displayed similarity to documented venom proteins (Additional file 1: Table S2). The set of reads lacking similarity to *Serpentes *entries was searched for the presence of cysteine-rich domains (eg. stretches of 50-100 amino acids containing ≥10% cysteine residues), as this feature is commonly shared by many toxin sequences [67]. The survey proved fruitless. Further attempts to enlarge the toxin dataset by searching specific databases, such as the Animal Toxin Database [68] or MEROPS [69], were also unsuccessful. The venom protein families identified, and their relative abundance, in the whole 454 read sequence dataset are listed in Table 1. The relative distribution of these venom protein families among the eight taxa investigated is shown in Table 2.

**Table 1:** Identity and relative abundance of venom protein entries identified in the whole 454 read sequence dataset of the 8 Costa Rican snake venom gland transcriptomes

	Number of reads	% of total venom protein entries
Bradykinin potentiating peptide (BPP)	9231	14.8
Cysteine-Rich Secretoy Peptide (CRISP)	1066	1.7
C-type lectin-like protein (CTL)	1039	1.6
Growth factor (GF)	789	1.2
L-amino acid oxidase (LAO)	2535	4.0
Phospholipase A_2 _(PLA_2_)	7065	11.3
Metalloproteinase (SVMP)	26646	42.7
Serine Proteinase (SP)	10019	16.0
5'-nucleotidase (5'-NTase)	374	0.6
Phosphodiesterase (PDE)	119	0.2
Glutaminyl cyclase (GC)	170	0.3
Cobra Venom Factor (CVF)	8	0.01
Crotamine (CRO)	22	0.04
Sarafotoxin (SARA)	3	0.005
Waprin (WAP)	26	0.04
Kunitz-type inhibitor (KUN)	21	0.03
Kazal-type inhibitor (KAZ)	21	0.03
Hyaluronidase (HYA)	24	0.04
Ohanin (OHA)	2412	3.9
Three-Finger Toxin (3FTx)	845	1.3

Their relative abundance in each transcriptome is displayed in Table 2.

**Table 2:** Relative contribution of the different venom protein family hits in each of the Costa Rican snake venom gland transcriptome

	*C. simus*		*B. asper *(Car)		*B. asper *(Pac)		*C. godmani*		*A. picadoi*		*A. mexicanus*		*B. schlegelii*		*B. lateralis*	
	Reads	%	Reads	%	Reads	%	Reads	%	Reads	%	Reads	%	Reads	%	Reads	%
BPP	178	13.4	4232	15.0	169	12.2	818	8.3	1634	16.4	406	13.0	444	15.3	1350	23.6
CRISP	0	0	245	0.8	3	0.2	253	2.6	261	2.6	17	0.5	105	3.6	182	3.2
CTL	32	2.4	287	1.0	19	1.3	23	0.2	476	4.8	15	0.5	7	0.2	180	3.1
GF	60	4.5	275	0.9	38	2.7	64	0.6	56	0.6	50	1.6	142	4.9	103	1.8
LAO	50	3.8	1197	4.2	33	2.4	537	5.5	308	3.1	121	3.9	92	3.2	197	3.4
PLA_2_	161	12.1	5026	17.8	195	14.2	777	7.9	228	2.3	224	7.2	278	9.6	176	3.0
SVMP	86	6.5	11733	41.6	559	40.6	4144	42.2	5583	56.1	1204	38.7	762	26.3	2575	44.9
SP	373	28.1	3790	13.4	114	8.3	2770	28.1	1089	10.9	692	22.2	594	20.5	597	10.4
5'-NTase	3	0.2	214	0.7	3	0.2	67	0.7	13	0.1	27	0.8	5	0.17	42	0.7
PDE	3	0.2	56	0.2	3	0.2	13	0.1	8	0.08	0	0	2	0.06	33	0.6
GC	5	0.4	108	0.4	4	0.3	32	0.3	13	0.1	7	0.2	0	0	1	0.02
CVF	2	0.15	2	0.006	0	0	1	0.01	0	0	2	0.06	1	0.03	0	0
CRO	0	0	10	0.03	4	0.3	2	0.02	4	0.04	0	0	1	0.03	1	0.02
SARA	1	0.07	2	0.006	0	0	0	0	0	0	0	0	0	0	0	0
WAP	0	0	26	0.09	0	0	0	0	0	0	0	0	0	0	0	0
KUN	2	0.15	10	0.03	3	0.2	0	0	1	0.01	0	0	4	0.12	1	0.02
KAZ	0	0	0	0	0	0	0	0	0	0	0	0	9	0.3	12	0.2
HYA	3	0.2	7	0.02	1	0.07	4	0.04	1	0.01	1	0.03	5	0.17	2	0.03
OHA	199	15.0	779	2.8	165	11.9	256	2.	233	2.3	254	8.2	338	11.6	188	3.3
3FTx	169	12.7	221	0.8	65	4.7	63	0.6	43	0.4	89	2.8	104	3.6	91	1.6

Protein family names are abbreviated as in Table 1.

Among the proteins listed in Table 1, glutaminyl cyclase (GC) belongs to the group of venom proteins without demonstrated toxic activity. Glutaminyl cyclase has been been identified in the venom proteomes of *B. jararaca*, *C. atrox*, and *C. durissus terrificus *[70-72]. N-terminal pyrrolidone carboxylic acid (pyroglutamate, pGlu) formation from its glutaminyl (or glutamyl) precursor is required in the maturation of numerous bioactive peptides. Snake venom GC is likely involved in the biosynthesis of pyroglutamyl peptides such as hypotensive BPPs [73,74], and endogenous inhibitors of metalloproteinases, pEQW, pENW, and pEKW [75,76]. Accumulation of peptide inhibitors in venoms provides a basis for attenuating the proteolytic activity of venom gland-stored SVMPs, preventing thereby autodigestion [77]. Mature PIII-SVMPs secreted into the venom proteome usually contain an N-terminal pyroglutaminyl residue (unpublished results), suggesting the action of the glutaminyl cyclase downstream of the proteolytic processing of the metalloproteinase precursor. However, the structural/functional consequences of N-terminal cyclation are unknown.

To estimate the number of toxin transcript sequences expressed in each transcriptome, multiple alignments of all reads clustered in the same protein family were generated, using the most similar full-length reference sequence as template. It was then realized that a large number of "*Serpentes *venom protein" reads did not align with translated ORFs. Instead, these reads appeared to represent 5'UTR, 3'UTR, and microsatellite loci. Particularly, all reads matching "cobra venom factor", "crotamine", "crotasin", and "sarafotoxin" entries corresponded to non-translated, mostly (87-100%) microsatellite DNA. In addition, 2397 out of 2412 reads for ohanin, and 840/845 3FTx reads aligned with microsatellite DNA. Microsatellite sequences accounted also for 66% GF, 49% SP, 36% PLA_2_, 27% CRISP, 15% LAO, and 8% CTL, but represented less than 5% of the reads of the rest of venom protein classes listed in Table 2 and Additional file 1: Table S3. On the other hand, the bulk (> 99%) of non-microsatellite untranslated sequences corresponded to 3' UTRs. Additional file 1: Table S3 summarizes the number of reads aligned to translated regions of reference snake venom toxin sequences. The occurrence of a large number of microsatellites in the venomous snake *Agkistrodon contortrix *has been recently reported by Castoe and colleagues [78], who used the 454 Genome Sequencer FLX next-generation sequencing platform to sample randomly ~27 Mbp (128,773 reads) of the genome of this species. These authors identified microsatellite loci in 11.3% of all reads obtained, with 14612 microsatellite loci identified in total.

The presence of mRNA coding for 3'-untranslated regions of toxins points to a) a bias due to the first-strand synthesis method used, which produced cDNA libraries enriched in 3'-end-transcripts, b) incompletely sequenced transcripts or c) to transcription of nonfunctional gene copies. Relevant to the latter possibility, excepting *A. mexicanus *venom, which contains a small amount (<0.1%) of a 3Ftx [50], CVF, SARA, OHA, WAP, 5'NTase, PDE, GC, KUN, HYA and 3FTx have not been detected in the venom proteomes of the Costa Rican snakes sampled here [49-52] (Additional file 1: Table S4). Fry and colleagues [79] have shown that the venom system is a basal characteristic of the advanced snakes, and have investigated the timing of toxin recruitment events and patterns of toxin diversification across the full range of the ~100 million-year-old advanced snake clade [2,3,5,6,79]. These studies revealed single early recruitment events for each toxin type, including those identified here (Table 1), indicating that the venomous function arose once in squamate reptile evolution, at about 200 Myr ago. Structural and functional diversification of the venom system is best described by the birth-and-death model of protein evolution [80,81]. Pseudogenes in Costa Rican pitviper venom transcriptomes may thus represent relics of the evolution of their venom arsenal.

**Figure 1:**
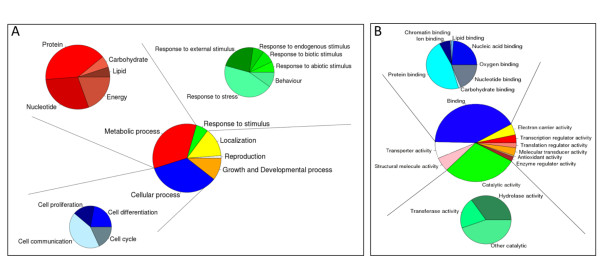
**Gene Ontology annotation of the non-toxin *Serpentes *reads according to their presumed biological process (panel A) and molecular function (panel B)**. The figure represents the combination of the reads from all eight species. However, each species transcriptome exhibited similar relative expression levels of GO-annotated non-toxin transcript classes.

The 37,961 reads comprising non-venom-protein BLAST hits were classified based on the presumed biological process to which they may contribute (Figure 1A) and on their putative molecular function (Figure 1B), according to the Gene Ontology database [48]. Their relative abundance, biological processes (general metabolism, response to external stimuli, cell differentiation, proliferation, and communication, cell cycle...), and molecular functions (transcription and translation, protein binding, catalysis, etc.) identified in this work generally agree with the broad categories reported for other viperid transcriptomes [27,32,52,53,82-84], and will not be described here in detail again. The most abundant transcripts are related to DNA transcription, mRNA translation, and post-translational processing of proteins, reflecting the high specialization of this tissue for expressing and secreting toxins to the lumen of the venom gland. Furthermore, many venom toxins bear a high number of cysteinyl residues, which are engaged in extensive intra- and intermolecular disulphide crosslinking [20]. Venom proteins such as disintegrins, C-type lectin-like proteins, serine proteinases, PLA_2_s, 3FTxs, and SVMPs occur in different oligomerization states [85-88]. The large structural impact at low energy cost of engineering disulphide bonds represents an opportunity for structural (and functional) diversification of proteins during evolution. Not surprisingly, protein disulphide isomerase (PDI), an enzyme and chaperone involved in disulphide bond formation in the endoplasmic reticulum [89,90], represents a highly expressed gene transcript (1859 reads; 5.1% of non-venom-protein reads) in all venom gland transcriptomes, ranging from 2.3% in *C. simus *to 6.9% in *B. lateralis*.

It is also worth mentioning the finding of reads for ribosomal 12S and 16S RNAs. This finding suggests that either internal mRNA A-rich tracts may have acted as priming sites in the cDNA synthesis, or that these messengers contained poly(A) tails. The possibility that rRNAs represent some residual contamination in the mRNA preparation should also be taken into account. Although polyadenylation is a distinctive feature of mRNA, polyadenylation of rRNA has been reported to occur in mammals and several unicellular organisms (*Candida albicans*, *Saccharomyces cerevisiae*, *Leishmania braziliensis *and *L. donovani*), and it may have a quality control role in rRNA degradation [91-94]. Polyadenylated ribosomal RNA has been also reported in the venom gland transcriptome of the Desert Massasauga Rattlesnake (*Sistrurus catenatus edwardsii*) [95].

### Calculation of the minimum number of gene copies from each toxin family

**Figure 2:**
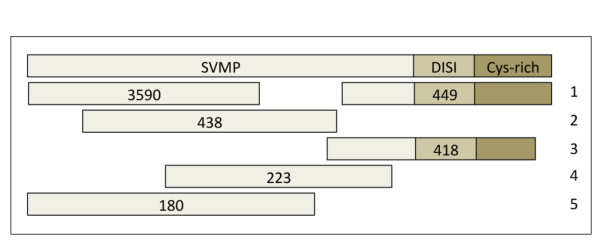
**Calculation of the minimum number of gene copies**. Multiple alignment of the top six SVMP transcripts of *B. asper *(Car) (Additional file 1: Table S6) using the sequence (top) of the most similar database-annotated toxin sequence as template. Each line of the multiple sequence alignment displays a distinct set of contig(s), comprised by a unique set of reads indicated in parentheses (see also Additional file 1: Table S5). Since the short average length of the reads and the low coverage of reads per contig prevented the assemblage of reliable gene sequences, each line of the alignment corresponds to at least a distinct gene of the SVMP multigene family translated into the venom gland transcriptome of *B. asper *(car).

**Figure 3:**
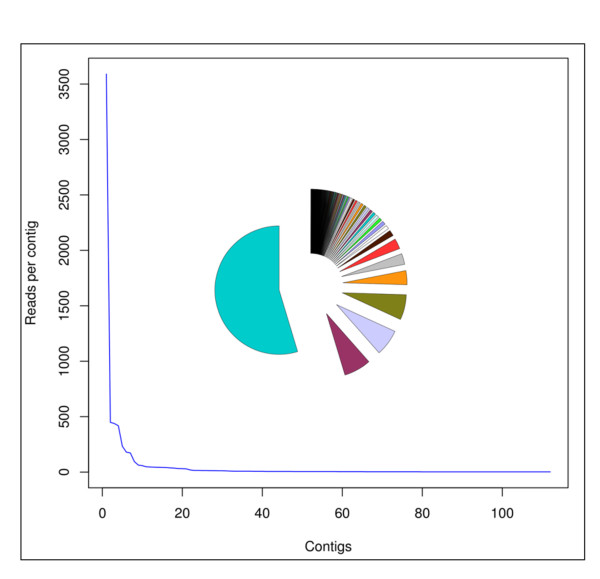
**Cartesian graph and corresponding chart pie displaying the uneven distribution of the number of reads per contig among the 29 SVMP genes identified in the venom gland transcriptome of *B. asper *(car)**.

An estimation of the minimum number of genes from each toxin family transcribed into the venom gland transcriptome of each species was calculated from the multiple alignments of reads matched to a full-length reference sequence of each toxin family (Figure 2). To this end, the nucleotide sequences of the ORF-coding reads of each venom protein family were assembled into contigs using MIRA and an iterative multiple-pass reference-guided protocol. MIRA is recommended for analysis of a normalized dataset or a filtered set of reads that did not have extreme coverage [64]. Each line of the multiple alignment (Figure 2) contained a distinct set of contigs spanning the maximum possible number of nucleotide positions of the reference sequence. Since the short average length of singletons and contigs (174-208 bp), and the low coverage of reads per contig (6), prevented the generation of full-length gene sequences, each line of the alignment corresponds to one or more synthetic gene. We considered two contigs as different if their nucleotide sequences depart in more number of positions than expected from a sequencing error rate of 1.35%, and the same mutated residues were found in at least two other reads. For each toxin family from each venom gland transcriptome a representation of the "number of reads per contig" vs. "number of contigs" was plotted, and only contigs accounting for ~95% of all assembled reads were considered. The rationale for introducing this quality trimming is because in this way only contigs in which the observed sequence differences were validated in a significant number of reads were taken into account, eliminating thus potential false positives due to sequencing errors, which generate "orphan" reads. The number of topologically equivalent homologous multiply-aligned reads corresponds thus to the minimum number of genes from a given toxin family transcribed into the venom gland transcriptome (Figure 2). The outcome of this analysis is displayed in Table 3. The estimated number of toxin-coding genes is in line with the number of different proteins identified in the respective venom proteomes: *C. simus *(27 reverse-phase HPLC fractions, ~20 proteins) [52]; *B.asper *(Car) (31 HPLC fractions, ~30 proteins) [51]; *B. asper *(Pac) (30 HPLC fractions, ~27 proteins) [51]; *A. mexicanus *(41 HPLC fractions) and *A. picadoi *(30 HPLC fractions), each containing bradykinin-potentiating peptides and around 25-27 proteins [50]; and *B. schlegelii *(34 HPLC fractions) and *B. lateralis *(34 HPLC fractions) matched to ~29 and 27 proteins, respectively [49]. Moreover, in most cases the overwhelming majority of reads of the large multigene toxin families (i.e., SVMP, PLA_2_, and SP) cluster into a small subset of contigs (Additional file 1: Tables S5, S6 and S7). The uneven distribution of SVMPs of *B. asper *(Car) (Figure 3) clearly illustrates this point: 3590 out of 6746 reads clustered into a single contig, and only 6 other contigs were assembled from 449-173 reads. The remaining 22 transcripts comprised each 95-12 reads. The low number of venom proteins inferred from our 454 transcriptomic analysis is also in concordance with a recent high-throughput venom pland transcriptomic analysis for the Eastern Diamond Rattlesnake (*C. adamanteus*), which identified 40 unique toxin transcript [40]. The most diverse and highly expressed toxin classes were the SVMPs (11 isoforms), serine proteinases and C-type lectin-like proteins (9 different protein species each).

**Table 3:** Estimation of the minimum number of toxin family gene copies translated in the venom gland transcriptomes of Costa Rican snakes

	*C. simus*	*B. asper *(Car)	*B. asper *(Pac)	*C. godmani*	*A. picadoi*	*A. mexicanus*	*B. schlegelii*	*B. lateralis*
BPP	1	1	1	2	1	1	4	2
CRISP	0	2	1	2	4	1	2	1
CTL	2	5	2	3	9	1	0	5
GF	2	5	1	3	3	1	1	1
LAO	3	2	2	4	5	2	3	3
PLA_2_	3	9	4	4	2	2	1	3
SVMP	9	29	5	19	15	4	14	20
SP	6	15	1	13	7	6	8	11
5'-NTase	1	3	1	2	2	2	1	3
PDE	1	1	1	2	2	0	1	2
GC	1	2	1	1	1	1	0	1
WAP	0	2	0	0	0	0	0	0
HYA	2	2	0	1	1	1	0	1
OHA	0	0	0	0	1	1	0	0
3FTx	1	0	0	0	0	0	0	0
KUN	0	0	0	0	0	0	1	0
KAZ	0	0	0	0	0	0	1	1

Protein family names are abbreviated as in Table 1.

The insight provided by our present transcriptomic data, supported by previous proteomic studies, indicate that the venoms of the Costa Rican snakes investigated are comprised by toxins belonging to a few major protein families. In addition, our data suggest that different genes of a multigene family are subjected to very distinct transcription (and translation) yields, i.e. as the result of distinct stability and translational rates of the messengers.

### Comparison of transcriptomes and proteomes

**Figure 4:**
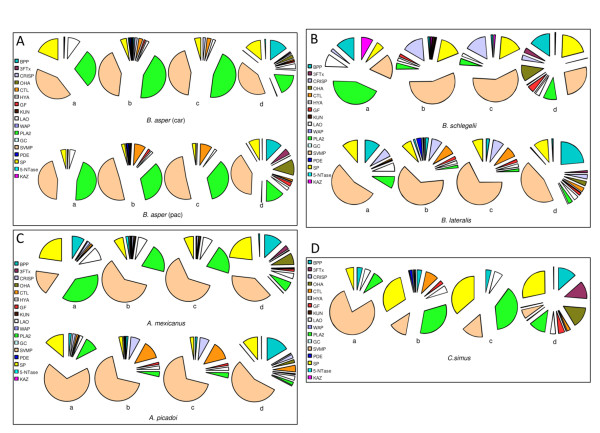
**Transcriptomes versus proteomes**. Comparison of the protein composition of the venom of Costa Rican snakes reported from proteomic analysis (chart pies labelled "**a**") (Additional file 1: Table S4)^49-52 ^and predicted from their venom gland transcriptomes (this work). Chart pies "**b**" display the relative occurrence of ORF-coding reads listed in Additional file 1: Table S4 normalized for the full-length DNA sequence of a canonical member of the protein family (%mol). Panels **c **show the relative abundance (mol%) of toxin families as in panels "**b**" but computing only toxins previously identified in the venom proteome^49-52^. Chart pies depicted in panels **d **show the relative composition (reads%) of all venom protein family hits in each of the Costa Rican snake venom gland transcriptome (Table 2). Protein family names are abbreviated as in Table 1.

The relative abundance of the different toxin families in each transcriptome was calculated as the percentage of toxin family-specific reads relative to all BLAST hits (Table 2) or to the set of reads aligned to translated (ORF) regions of a reference sequences (Additional file 1: Table S3). To estimate the relative contribution of each toxin family, the total number of nucleotides of the ORF-coding reads was normalized for the full-length nucleotide sequence of a canonical member of the protein family. When available, the obtained figures were compared with the percentages of toxin families reported for the venom proteome of the same species. The outcome of this comparative analysis is compiled in Additional file 1: Tables S3 and S4. Strong discrepancies between the transcriptome-computed and the proteome-gathered toxin compositions are obvious at first sight. The best, although still far from perfect, agreement between proteomic and transcriptomic data occured when the relative abundance of transcripts was computed using all the reads (ORFs + UTRs) belonging only to toxin classes detected in the venom proteome (Figure 4). This would support the view that the majority of reads matching UTRs may indeed form part of parent translatable mRNAs. However, particularly *B. asper *(Car) (Figure 4A), *B. schlegelii *(Figure 4B), *A. mexicanus *(Figure 4C), and *C. simus *(Figure 4D) strongly depart from this picture. The reasons underlying this discrepancy are elusive, since no clear trend within or between species is apparent, but both intrinsic (methodological) and extrinsic (biological) factors may be involved. Hence, besides the difficulty of deciding between bias due to cDNA libraries enriched in 3'-end-transcripts, and the presence of transcripts of pseudogenes in the transcriptome, we hypothesize that the distinct stability and translational rates of the messengers might also contribute to the observed differences between transcriptome and proteome. Thus, a high abundant messenger subjected to a higher degradation rate may produce the same concentration as a low abundance but more stable mRNA or exhibiting a higher translational rate. The observation that *B. asper *(Car) and *B. asper *(Pac), as well as *A. mexicanus *and *A. picadoi*, exhibit highly similar transcriptomes but strongly depart in the relative toxin composition of their venom proteomes (Figure 4), indicates that individual mRNA species are translationally controlled in a species-dependent manner. The same conclusion can be drawn by comparing the proteome and transcriptome of *C. simus *(Figure 4D). In this respect, mounting evidences in yeast, indicate that the loading of ribosomes onto individual mRNA species varies broadly across a cellular transcriptome, and this finding is consistent with each transcript having a uniquely defined efficiency of translation [96-99].

**Figure 5:**
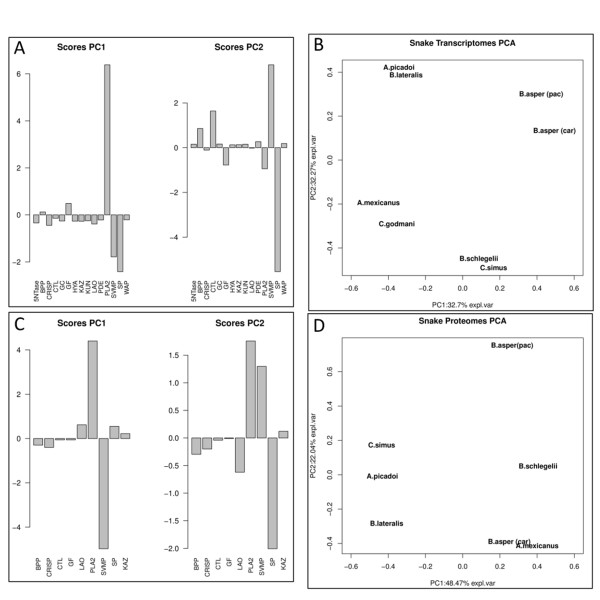
**Principal Component Analysis (PCA) of the Costa Rican snake venom gland transcriptomes (A, B) and the corresponding proteomes (C, D)**. Panels A and C show, respectively, the contributions to PC1 and PC2 of the different toxin families of the transcriptomes and the proteomes. Panels **B **and **D**, score plots displaying the segregation of the transcriptomes (B) and the proteomes (D) into different categories. In **B**, PC1 and PC2 contribute equally and together explain 65% of the observed transcriptome variability; in **D**, PC1 and PC2 explain 70% of the variability among proteomes.

A rough comparison of the transcriptomes (Table 2) shows that some toxin families are relatively constantly expressed among snakes while others exhibit greater variability. Principal Component Analysis (PCA) revealed that the abscissa (PC1) and the ordinate (PC2) each explained 32% of the observed variability (Figure 5A). PCA discriminated the eight transcriptomes into four groups (Figure 5B). Referring to the average value of the toxin family, the transcriptomes of *A. picadoi *and *B. lateralis *contain higher content (⇑) of SVMP reads and lower number (⇓) of PLA_2 _and SP reads; the two *B. asper *taxa express ⇑SVMPs and PLA_2_s and ⇓ SPs; *A. mexicanus *and *C. godmani *contain ⇑SVMPs, ⇑SPs, and slightly ⇓PLA_2_s; and *B. schlegelii *and *C. simus *contain ⇓SVMPs, ⇑SPs, and average number (⇔) of PLA_2 _reads. These data point to convergent and divergent evolutionary trends among pitvipers. PCA of the venom proteomes listed in Additional file 1: Table S4 revealed different clustering of taxa (Figure 5, panels C and D), and unequal contributions of PC1 (48%) and PC2 (22%) to venom variability. These results illustrate the versatility of snake venoms as a system to achieve the purpose of subduing prey through different strategies [100]. On the other hand, the lack of any apparent correlation between the PCA score plots for transcriptome (Figure 5B) and proteome (Figure 5D) data, further highlights the existence of variable translational patterns across species. Clearly, our results emphasize the relevance of combining detailed proteomic and transcriptomic studies for a thorough characterization of the venom toxin repertoire and the factors regulating transcription and translation.

In a previous proteomic study, we identified two RP-HPLC fractions of *B. schlegelii *venom as Kazal-type proteinase inhibitor-like proteins (family PD000417 in the ProDom database, http://prodom.prabi.fr/prodom/current/html/home.php) [49]. Kazal-type inhibitor-like proteins (KAZ) have not been found in any other snake venom reported to date, casting doubts on their venom gland origin, on the one hand, or pointing to a recruitment event of these proteins along the speciation of the Neotropical pitviper clade [49]. Now we report the finding of 9 and 12 reads encoding ORF regions of KAZ in *B. schlegelii *and *B. lateralis *transcriptomes, respectively (Table 2 and Additional file 1: Table S4). In each species, all KAZ reads assembled into a single transcript (Table 3), suggesting a monogenic origin. The occurrence of Kazal-type inhibitor-like proteins only in the venom gland transcriptomes of the two Bothriechis taxa (Additional file 1: Tables S4 and S5) supports the view of a genus-specific recruitment event during the early-Middle Miocene ~14 Mya, the estimated divergence time for Bothriechis in a model of Middle American highland speciation [101]. On the other hand, the presence of Kazal-type proteins in the venom proteome of *B. schlegelii*, the basal species of the Bothriechis clade [102], suggests a species-specific expression of this class of protein. Though a number of Kazal-like domains harbor serine proteinase inhibitor activity, these protein scaffolds are also present in the extracellular part of a number of proteins, which are not known to be proteinase inhibitors. Clearly, further investigations are needed to assess the biological activity of the Kazal-type proteins, and the role that these proteins may have played in the early adaptive radiation of the *Bothriechis *clade.

### Transcriptome-based cladistic analysis of the Costa Rican snake venom gland transcriptomes

**Figure 6:**
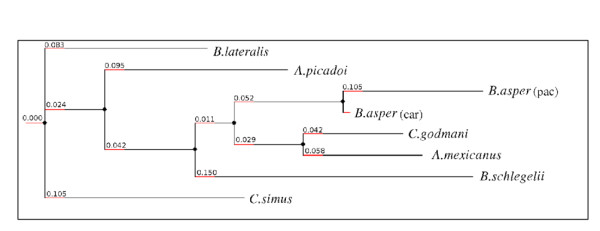
**Cladogram of phylogenetic alliances among Central American snakes inferred from comparison of concatenated consensus sequences for SVMP, SP, BPP, LAO and PLA_2 _gathered from analysis of their venom gland transcriptomes**. Numbers at branching points indicate the degree of sequence divergence (0.1 = 10% sequence divergence).

To assess structural relationships between the transcriptomes, consensus sequences were constructed for those major toxin families shared by all snake venom gland transcriptomes, i.e. BPP, LAO, PLA2, SVMP, and SP. To assess the degree of kinship between the Costa Rican snake venom gland transcriptomes, species-specific synthetic sequences were generated by the concatenation of the 5 toxin-family consensus sequences (in the order described above), and using these synthetic sequences as input, a cladogram was built using the suite of web-tools Phylemon (http://phylemon.bioinfo.cipf.es) (Figure 6). Mutation is the driving force of evolution, but inferring evolutionary distances from multiple sequence alignments can yield misleading results if the mutation rates of the genes being compared are unequal across species. Given knowledge of the degree of mutation rate heterogeneity, appropriate algorithms can be applied to correct unbiasness and inaccuracy of the phylogenetic reconstruction [103-105]. Globally, translated assembled sequences displayed mean variability levels between 0 and 7.4% (computed as number of variable residues divided by sequence length), being the SVMPs and SPs the toxin families which accumulate more amino acid substitutions. Although the cladogram depicted in Figure 6 should not be regarded as an evolutionary hypothesis, the divergence of the Atropoides taxa, and the clustering of *A. mexicanus *and *C. godmani *deserves discussion.

Despite the efforts of numerous authors, phylogenetic relationships within the subfamily Crotalinae remain controversial, particularly at the intergeneric level [100,105]. In particular, several analyses, even from the same research group, support different phylogenetic models. Thus, Bayesian Markov chain Monte-Carlo results suggested the monophyly of the three genera of the Porthidium group (*Atropoides*, *Cerrophidion*, and *Porthidium*) and indicated that *Cerrophidion *and *Porthidium *form a clade that is the sister taxon to *Atropoides *[106]. On the other hand, genus *Atropoide*s has been also inferred through Bayesian phylogenetic methods to be paraphyletic with respect to *Cerrophidion *and *Porthidium*, due to *Atropoides picadoi *being distantly related to other *Atropoides *species [107,108]. Although resolving the phylogenetic relationships among the Neotropical pitvipers of the Porthidium group requires a detailed genomic study of species occupying geographically close ecological niches, i.e., *Porthidium nasutum*, *Porthidium ophryomegas*, *Porthidium volcanicum*, and *Cerrophidion godmani*, both a previous proteomic study by Angulo and co-workers [50], who estimated that the similarity of venom proteins between the two Atropoides taxa may be around 14-16%, and the present transcriptomic analysis, support a large divergence between *A. mexicanus *and *A. picadoi*, and the closer kinship between *A. mexicanus *and *C. godmani*.

## Conclusions

The snake venom gland is a highly specialized and sophisticated organ, which harbors the cellular machinery that transformed throughout > 200 million years of evolution genes coding for ordinary proteins of disparate scaffolds, diverse ancestral bioactivities, and recruited from a wide range of tissues, into lethal toxins [2]. Although the details of recruitment and neofunctionalization of these proteins remain elusive, gene duplication events, followed by the accelerated evolution of some copies and degradation of others to pseudogenes, underlay the emergence of venom gland multigene toxin families. Comparative analysis of complete genome sequences of squamate reptiles would be extremely valuable for tracking the evolution of the venom system in lizards and snakes [5,6]. On the other hand, a deep understanding of the toxin gene expression pattern revealed by high-throughput transcriptomic may reveal taxon-specific adaptations and the underlying biological processes governing the formulation of the venom arsenal. In this respect, invoking a reverse venomics approach, knowledge of the end product of transcriptome translation, the venom proteome, may provide hints on the translation efficiency of toxin-coding transcripts. The high-throughput capability of next-generation sequencing technologies offer the opportunity to generate large transcriptome databases relatively rapidly, which may help to speed up the tedious, often *de novo *[20], assignment of proteomic-gathered data. Furthermore, analysis of the venom gland transcriptome enhances the comprehensibility of the venom proteome, and this in turn contributes to a more accurate interpretation of the transcriptome. The application of NGS to the analysis of snake venom transcriptomes, may represent the tool for opening the door to systems venomics.

## Methods

### Snake venom gland cDNA synthesis and sequencing

Venom glands were removed 3 days after venom milking, when transcription is maximal [109], from anesthetized snakes using fine forceps and immediately placed in RNAlater™ solution (Qiagen). 30 mg of tissue were disrupted and homogenized by a rotor-stator homogenizer, and total RNA was isolated using RNeasy Mini kit (Qiagen), quantified in a spectrophotometer, and quality-checked on an agarose gel discerning the 28S and 18S bands of ribosomal RNA. First strand cDNA was synthesized using RevertAid™ H Minus First Strand cDNA Synthesis Kit (Fermentas), which selectively transcribes full-length polyadenylated mRNA. The manufacturer's recommendations were followed except where specified. Approximately 5 μg of total RNA was used as starting material. In order to avoid polymerase slippage, a modified 3' 54-mer adaptor (5' GAGCTAGTTCTGGAG(T)_16_VN), which includes a type IIs enzyme (GsuI) site (underlined), was used for first-strand synthesis. This modified oligonucleotide effectively converts the long run of adenosine residues at the polyA tail into a sequence that causes fewer problems for dideoxy sequencing chemistry, and thus the resulting cDNA libraries were enriched in 3'-end-transcripts. To avoid internal cuts, the cDNA was hemimethylated by adding 5-methyl-dCTP to the dNTPs mix. The first strand cDNA was used as template for second strand synthesis by *E. coli *DNA Polymerase I and RNase H. Double strand (ds) cDNA was precipitated with ethanol and the pellet was resuspended in 70 μL of nuclease-free water and subjected to enzymatic digestion with GsuI for 4 hours at 30°C. The enzyme was then inactivated at 65°C for 20 minutes and the digested cDNA was stored at -20°C. For 454 pyrosequencing, the GS FLX General Library Preparation Method Manual workflow (Roche Diagnostics) was followed. To this end, 3 μg of final non-normalized cDNA library were sheared by nebulization into small fragments. The fragment ends were polished and short A/B adaptors were ligated onto both ends, providing priming regions to support both emulsion amplification and the pyrosequencing process. A biotin tag on the B adaptor allowed immobilization of the dscDNA library fragments onto streptavidin-conjugated magnetic beads and the subsequent isolation of the library of single strand cDNA sequencing templates. Each of the eight cDNA libraries was tagged with a unique 10-base sequence (MID, Multiplex IDentifier) that is recognized by the sequencing analysis software, allowing for automated sorting of MID-containing reads. Barcoded libraries were simultaneously sequenced in a Genome sequencing FLX System (Roche Applied Science) at Life Sequencing S.L. (Parc Científic Universitat de Valencia, Paterna, Valencia, Spain; http://www.lifesequencing.com) using the method developed by Margulies et al. [110].,

### Bioinformatic processing of the 454 reads, identification of toxin transcripts, and quantitation of the expression levels of toxin families

Additional file 2: Figure S1 displays a scheme of the data analysis pipeline developed to identify sequences of toxin molecules by similarity search against nucleotide databases, and elaborate a reference-guide estimation of transcripts, which included available NGS algorithms and in-house scripts. To this end, interspersed repeats and low complexity DNA sequences were masked from the transcript reads using RepeatMasker (version 3.2.7) [111]. RepeatMasker is available from the Institute for Systems Biology (http://www.systemsbiology.org) addressing http://repeatmasker.org. The program makes use of Repbase, which is a service of the Genetic Information Research Institute (http://www.girinst.org). Repbase is a comprehensive database of repetitive element consensus sequences (update of 20 January 2009). Data and computational resources for the Pre-Masked Genomes page is provided courtesy of the UCSC Genome Bioinformatics group (http://genome.ucsc.edu). Masked reads were then searched against the non-redundant NCBI database (http://blast.ncbi.nlm.nih.gov, release of March 2009) and the UniProtKB/Swiss-Prot Toxin Annotation Program database (http://us.expasy.org/sprot/tox-prot), using BlastX and BlastN [112] algorithms, specifying a cut-off value of e-03 and BLOSUM62 as scoring matrix. Snake venom gland-specific transcripts were selected from best BLAST-hit descriptions identifying GenBank entries belonging to the taxonomic suborder *Serpentes*. This taxonomic group is represented by 37,070 records comprising entries from 357 different genera. The subset of reads exhibiting similarity to *Serpentes *sequences were further filtered using a list of keywords (including the acronyms of all known toxin protein families described so far [20,25]) to distinguish putative snake venom toxins from non-toxin (ribosomal, mitochondrial, nuclear, etc.), ordinary proteins. In a second round of filtering, non-matched sequences were searched for structural features (eg. high cysteine content) expected for a putative toxin molecule. Non-toxin-assigned transcripts were functionally annotated using the Blast2GO software [113] and classified using GO-terms [114]. The relative expression of a given toxin protein family (mol%) was calculated as the number of reads assigned to this protein family (R_i_) normalized by the length (in nucleotides) of the reference transcript sequence (ntREF) and expressed as the % of total reads in the snake transcriptome (∑Reads): mol% toxin family "i" = %[(R_i_/ntREF)/∑Reads). The relationship between the expression levels of the different protein families in the different species sampled was analyzed by Principal Component Analysis (PCA). When possible, toxin transcriptome profiles were compared with available proteomic-based venom toxin profiles [115-118].

### Assessing molecular diversity within toxin families and cladistic analysis

To assess the molecular diversity within each toxin family, the phylogenetically nearest top-hit sequence was designated as the reference sequence for aligning all the toxin family-specific reads. To this end, each toxin family read was translated into the 6 possible reading frames and blasted against the reference protein sequence using tBlastN. Matched frames exhibiting e-value thresholds better than e-03 were aligned onto the reference sequence to create a multiple alignment using COBALT [119]. The multiple alignment was then parsed to create an assembled (consensus) toxin sequence in which each amino acid position is supported by at least four reads, representing at least 30% of the total number of reads at that position. Non-sequenced positions or those that did not meet the minimum coverage condition, are depicted in lower case in the assembled sequences. Positions where two or more amino-acids fulfilled the selection criteria were annotated as variable residues suggesting the occurrence of different alleles (isoforms) of the protein. Multiple sequence alignments based on transcriptomic data were computed with program MUSCLE [120] (version 3.52) using species-specific synthetic sequences constructed by the concatenation of the consensus sequences of the major toxin families shared by all snake venom gland transcriptomes, i.e. BPP, LAO, PLA2, SVMP, and SP, in this order. Phylogenetic tree reconstruction was done using the suite of web-tools Phylemon (http://phylemon.bioinfo.cipf.es/cgi-bin/tools.cgi) using the MUSCLE, ProtDist and Neighbor options of Phylip (version 3.65).

## Authors' contributions

PJ, MF-D, AA-G, and MS dissected the venom glands and construct the cDNA libraries. JD, along with JMG, AC, LS, and JJC, analyzed the transcriptomic data and participated in data interpretation and discussion, as well as in revising the article drafted by JJC. JD provided bioinformatic tools and lab space for conducting this study. All authors have and approved the final manuscript.

## Supplementary Material

Additional file 1 **Table S1**: RepeatMasker usage results and features of the sequence elements masked in the 8 Costa Rican venom gland transcriptomes analyzed **Table S2**: Summary of the 454 sequencing statistics and annotation of transcripts in the 8 venom gland transcriptomes. **Table S3**: Number of reads aligned to translated (ORF) regions of reference snake venom toxin sequences. **Table S4**: Relative occurrence (in %) of the ORF-coding reads listed in Table S3. **Table S5**: Distribution of reads per contig among the SVMP genes. **Table S6**: Distribution of reads per contig among the PLA_2 _genes. **Table S7**: Distribution of reads per contig among the serine proteinase genes. Click here for file

Additional file 2 **Figure S1**. Summary of the strategy employed to assembly and analyze the 454 pyrosequencing reads from the venom gland transcriptomes of the Costa Rican snakes *Bothrops asper *(from Caribbean and Pacific populations), *Bothriechis lateralis*, *Bothriechis schlegelii*, *Atropoides picadoi*, *Atropoides mexicanus*, *Crotalus simus*, and *Cerrophidion godmani*. Click here for file

## References

[B1] Mackessy SP Morphology and ultrastructure of the venom glands of the northern Pacific rattlesnake *Crotalus viridis oreganus* J Morphol 1991 208 109 128 10.1002/jmor.1052080106 29865511

[B2] Fry BG Wüster W Assembling an arsenal: origin and evolution of the snake venom proteome inferred from phylogenetic analysis of toxin sequences Mol Biol Evol 2004 21 870 883 10.1093/molbev/msh091 15014162

[B3] Fry BG Scheib H van der Weerd L Young B McNaughtan J Ramjan SF Vidal N Poelmann RE Norman JA Evolution of an arsenal: structural and functional diversification of the venom system in the advanced snakes (Caenophidia) Mol Cell Proteomics 2008 7 215 246 17855442 10.1074/mcp.M700094-MCP200

[B4] Vonk FJ Admiraal JF Jackson K Reshef R de Bakker MAG Vanderschoot K van den Berge I van Atten M Burgerhout E Beck A Mirtschin PJ Kochva E Witte F Fry BG Woods A Richardson MK Evolutionary origin and development of snake fangs Nature 2008 454 630 633 10.1038/nature07178 18668106

[B5] Fry BG Vidal N Norman JA Vonk FJ Scheib H Ramjan SF Kuruppu S Fung K Hedges SB Richardson MK Hodgson WC Ignjatovic V Summerhayes R Kochva E Early evolution of the venom system in lizards and snakes Nature 2006 439 584 588 10.1038/nature04328 16292255

[B6] Fry BG Vidal N van der Weerd L Kochva E Renjifo C Evolution and diversification of the Toxicofera reptile venom system J Proteomics 2009 72 127 136 10.1016/j.jprot.2009.01.009 19457354

[B7] Greene HW Dietary correlates of the origin and radiation of snakes Am Zool 1983 23 431 441

[B8] World Health Organization Rabies and envenomings. A neglected public health issue: Report of a consultative meeting 2007 Geneva: WHO http://www.who.int/bloodproducts/animal_sera/Rabies.pdf

[B9] Kasturiratne A Wickremasinghe AR de Silva N Gunawardena NK Pathmeswaran A Premaratna R Savioli L Lalloo DG de Silva HJ The global burden of snakebite: a literature analysis and modelling based on regional estimates of envenoming and deaths PLoS Med 2008 5 e218 10.1371/journal.pmed.0050218 18986210 PMC2577696

[B10] Chippaux JP Goyffon M Epidemiology of scorpionism: a global appraisal Acta Trop 2008 107 71 79 10.1016/j.actatropica.2008.05.021 18579104

[B11] Koh DCI Armugam A Jeyaseelan K Snake venom components and their applications in biomedicine Cell Mol Life Sci 2006 63 3030 3041 10.1007/s00018-006-6315-0 17103111 PMC11135979

[B12] Calvete JJ Venomics: digging into the evolution of venomous systems and learning to twist nature to fight pathology J Proteomics 2009 72 121 126 10.1016/j.jprot.2009.01.018 19457346

[B13] Harvey AL Bradley KN Cochran SA Rowan EG Pratt JA Quillfeldt JA Jerusalinsky DA What can toxins tell us for drug discovery? Toxicon 1998 36 1635 1640 10.1016/S0041-0101(98)00156-1 9792180

[B14] Ménez A Functional architectures of animal toxins: a clue to drug design? Toxicon 1998 36 1557 1572 10.1016/S0041-0101(98)00148-2 9792172

[B15] Ménez A Stöcklin R Mebs D "Venomics" or: the venomous systems genome project Toxicon 2006 47 255 259 10.1016/j.toxicon.2005.12.010 16460774

[B16] Escoubas P King GF Venomics as a drug discovery platform Expert Rev Proteomics 2009 6 221 224 10.1586/epr.09.45 19489692

[B17] Vetter I Davis JL Rash LD Anangi R Mobli M Alewood PF Lewis RJ King GF Venomics: a new paradigm for natural products-based drug discovery Amino Acids 2011 17 15 28 10.1007/s00726-010-0516-4 20177945

[B18] Gutiérrez JM León G de Lima ME, Pimenta AMC, Martin-Euclaire MF, Zingali RB, Rochat H Snake antivenoms: Technological, clinical and public health issues Animal Toxins: State of the Art. Perspectives in Health and Biotechnology 2009 Belo Horizonte: Editora UFMG 393 421

[B19] Espino-Solis GP Riaño-Umbarila L Becerril B Possani LD Antidotes against venomous animals: state of the art and prospectives J Proteomics 2009 72 183 199 10.1016/j.jprot.2009.01.020 19457345

[B20] Calvete JJ Juárez P Sanz L Snake venomics. Strategy and applications J Mass Spectrom 2007 42 1405 1414 10.1002/jms.1242 17621391

[B21] Fox JW Serrano SM Exploring snake venom proteomes: multifaceted analyses for complex toxin mixtures Proteomics 2008 8 909 920 10.1002/pmic.200700777 18203266

[B22] Escoubas P Quinton L Nicholson GM Venomics: unravelling the complexity of animal venoms with mass spectrometry J Mass Spectrom 2008 43 279 295 10.1002/jms.1389 18302316

[B23] Dos Santos LD Santos KS Pinto JR Dias NB de Souza BM dos Santos MF Perales J Domont GB Castro FM Kalil JE Palma MS Profiling the proteome of the venom from the social wasp *Polybia paulista*: a clue to understand the envenoming mechanism J Proteome Res 2010 9 3867 3877 10.1021/pr1000829 20540563

[B24] Rodríguez de la Vega RC Schwartz EF Possani LD Mining on scorpion venom biodiversity Toxicon 2009 56 1155 1161 19931296 10.1016/j.toxicon.2009.11.010

[B25] Junqueira de Azevedo ILM Diniz MRV Ho PL de Lima ME, Pimenta AMC, Martin-Euclaire MF, Zingali RB, Rochat H Venom gland transcriptomic analysis Animal Toxins: State of the Art. Perspectives in Health and Biotechnology 2009 Belo Horizonte: Editora UFMG 693 713

[B26] Sanz L Escolano J Ferretti M Biscoglio MJ Rivera E Crescenti EJ Angulo Y Lomonte B Gutiérrez JM Calvete JJ Snake venomics of the South and Central American Bushmasters. Comparison of the toxin composition of *Lachesis muta *gathered from proteomic versus transcriptomic analysis J Proteomics 2008 71 46 60 10.1016/j.jprot.2007.10.004 18541473

[B27] Wagstaff SC Sanz L Juárez P Harrison RA Calvete JJ Combined snake venomics and venom gland transcriptomic analysis of the ocellated carpet viper, *Echis ocellatus* J Proteomics 2009 71 609 623 10.1016/j.jprot.2008.10.003 19026773

[B28] Fasoli E Sanz L Wagstaff S Harrison RA Righetti PG Calvete JJ Exploring the venom proteome of the African puff adder, *Bitis arietans*, using a combinatorial peptide ligand library approach at different pHs J Proteomics 2010 73 932 942 10.1016/j.jprot.2009.12.006 20026262

[B29] Ho PL Soares MB Yamane T Raw I Reverse biology applied to *Micrurus corallinus*, a south american coral snake J Toxicol Toxin Reviews 1995 14 327 337

[B30] Adams MD Kelley JM Gocayne JD Dubnick M Polymeropoulos MH Xiao H Merril CR Wu A Olde B Moreno RF Kerlavage AR McCombie WR Venter JC Complementary DNA sequencing: expressed sequence tags and human genome project Science 1991 252 1651 1656 10.1126/science.2047873 2047873

[B31] Calvete JJ Marcinkiewicz C Sanz L Snake venomics of *Bitis gabonica gabonica*. Protein family, composition, subunit organization of venom toxins, and characterization of dimeric disintegrins Bitisgabonin-1 and Bitisgabonin-2 J Proteome Res 2007 6 326 336 10.1021/pr060494k 17203976

[B32] Valente RH Guimarães PR Junqueira M Neves-Ferreira AG Soares MR Chapeaurouge A Trugilho MR León IR Rocha SL Oliveira-Carvalho AL Wermelinger LS Dutra DL Leão LI Junqueira-de-Azevedo IL Ho PL Zingali RB Perales J Domont GB *Bothrops insularis *venomics: a proteomic analysis supported by transcriptomic-generated sequence data J Proteomics 2009 72 241 255 10.1016/j.jprot.2009.01.001 19211044

[B33] Chippaux JP Williams V White J Snake venom variability: methods of study, results and interpretation Toxicon 1991 29 1279 1303 10.1016/0041-0101(91)90116-9 1814005

[B34] Wagstaff SC Harrison RA Venom gland EST analysis of the saw-scaled viper, *Echis ocellatus*, reveals novel α_9_β_1 _integrin-binding motifs in venom metalloproteinases and a new group of putative toxins, renin-like aspartic proteases Gene 2006 377 21 32 16713134 10.1016/j.gene.2006.03.008

[B35] Wheat CW Rapidly developing functional genomics in ecological model systems via 454 transcriptome sequencing Genetica 2010 138 433 451 10.1007/s10709-008-9326-y 18931921

[B36] Linnarsson S Recent advances in DNA sequencing methods. General principles of sample preparation Exp Cell Res 2010 316 1339 1343 10.1016/j.yexcr.2010.02.036 20211618

[B37] Morozova O Hirst M Marra MA Applications of new sequencing technologies for transcriptome analysis Annu Rev Genomics Hum Genet 2009 10 135 151 10.1146/annurev-genom-082908-145957 19715439

[B38] Mardis ER The impact of next-generation sequencing technology on genetics Trends Genet 2008 24 133 141 10.1016/j.tig.2007.12.007 18262675 PMC2680276

[B39] Zhang B Liu Q Yin W Zhang X Huang Y Luo Y Qiu P Su X Yu J Hu S Yan G Transcriptome analysis of *Deinagkistrodon acutus *venomous gland focusing on cellular structure and functional aspects using expressed sequence tags BMC Genomics 2006 7 152 10.1186/1471-2164-7-152 16776837 PMC1525187

[B40] Rokyta DR Wray KP Lemmon AR Lemmon EM Caudle SB A high-throughput venom-gland transcriptome for the Eastern Diamond Rattlesnake (*Crotalus adamanteus*) and evidence for pervasive positive selection across toxin classes Toxicon 2011 57 657 671 10.1016/j.toxicon.2011.01.008 21255598

[B41] Wang Z Gerstein M Snyder M RNA-Seq: a revolutionary tool for transcriptomics Nat Rev Genet 2009 10 57 63 10.1038/nrg2484 19015660 PMC2949280

[B42] Gutiérrez JM Mackessy SP Snakebite envenomation in Central America Handbook of Venoms and Toxins of Reptiles 2009 CRC Press: Boca Raton, Florida 491 507

[B43] Campbell JA Lamar WW The Venomous Reptiles of the Western Hemisphere 2004 Cornell University Press: Ithaca

[B44] Wheeler DL Barrett T Benson DA Bryant SH Canese K Chetvernin V Church DM Dicuccio M Edgar R Federhen S Feolo M Geer LY Helmberg W Kapustin Y Khovayko O Landsman D Lipman DJ Madden TL Maglott DR Miller V Ostell J Pruitt KD Schuler GD Shumway M Sequeira E Sherry ST Sirotkin K Souvorov A Starchenko G Tatusov RL Tatusova TA Wagner L Yaschenko E Database resources of the National Center for Biotechnology Information Nucleic Acids Res 2008 36 Database D13 21 10.1093/nar/gkm1000 PMC2238880 18045790

[B45] Wang W Wang Y Zhang Q Qi Y Guo D Global characterization of *Artemisia annua *glandular trichome transcriptome using 454 pyrosequencing BMC Genomics 2009 10 465 10.1186/1471-2164-10-465 19818120 PMC2763888

[B46] McClintock B Controlling elements and the gene Cold Spring Harb Symp Quant Biol 1956 21 197 216 13433592 10.1101/sqb.1956.021.01.017

[B47] Bannert N Kurth R Retroelements and the human genome: new perspectives on an old relation Proc Natl Acad Sci USA 2004 101 14572 14579 10.1073/pnas.0404838101 15310846 PMC521986

[B48] Deininger PL Batzer MA Mammalian retroelements Genome Res 2002 12 1455 1465 10.1101/gr.282402 12368238

[B49] Hughes JF Coffin JM Evidence for genomic rearrangements mediated by human endogenous retroviruses during primate evolution Nat Genet 2001 29 487 489 10.1038/ng775 11704760

[B50] Jern P Coffin JM Effects of retroviruses on host genome function Annu Rev Genet 2008 42 709 732 10.1146/annurev.genet.42.110807.091501 18694346

[B51] Pi W Zhu X Wu M Wang Y Fulzele S Eroglu A Ling J Tuan D Long-range function of an intergenic retrotransposon Proc Natl Acad Sci USA 2010 107 12992 12997 10.1073/pnas.1004139107 20615953 PMC2919959

[B52] Junqueira-de-Azevedo ILM Ho PL A survey of gene expression and diversity in the venom glands of the pit viper snake *Bothrops insularis *through the generation of expressed sequence tags (ESTs) Gene 2002 299 279 291 10.1016/S0378-1119(02)01080-6 12459276

[B53] Junqueira-de-Azevedo ILM Ching ATC Carvalho E Faria F Nishiyama MY Jr Ho PL Diniz MRV *Lachesis muta *(Viperidae) cDNAs reveal diverging pit viper molecules and scaffolds typical of cobra (Elapidae) venoms: implications for snake toxin repertoire evolution Genetics 2006 172 877 889 10.1534/genetics.106.056515 PMC1526512 16582429

[B54] Ching AT Rocha MM Leme AFP Pimenta DC Furtado MFD Serrano SM Ho PL Junqueira de Azevedo IL Some aspects of the venom proteome of the Colubridae snake *Philodryas olfersii *revealed from a Duvernoy's (venom) gland transcriptome FEBS Lett 2006 580 4417 4422 10.1016/j.febslet.2006.07.010 16857193

[B55] Kordis D Gubenšek F Bov-B long interspersed repeated DNA (LINE) sequences are present in *Vipera ammodytes *phospholipase A2 genes and in genomes of Viperidae snakes Eur J Biochem 1997 246 772 779 10.1111/j.1432-1033.1997.00772.x 9219538

[B56] Kordis D Gubenšek F The Bov-B lines found in *Vipera ammodytes *toxic PLA2 genes are widespread in snake genomes Toxicon 1998 36 1585 1590 10.1016/S0041-0101(98)00150-0 9792174

[B57] Ikeda N Chijiwa T Matsubara K Oda-Ueda N Hattori S Matsuda Y Ohno M Unique structural characteristics and evolution of a cluster of venom phospholipase A_2 _isozyme genes of *Protobothrops flavoviridis *snake Gene 2010 461 15 25 10.1016/j.gene.2010.04.001 20406671

[B58] Ohno M Evolution by gene duplication 1970 Springer Verlag: Berlin

[B59] Lynch VJ Inventing an arsenal: adaptive evolution and neofunctionalization of snake venom phospholipase A_2 _genes BMC Evol Biol 2007 7 2 10.1186/1471-2148-7-2 17233905 PMC1783844

[B60] Gibbs HL Rossiter W Rapid evolution by positive selection and gene gain and loss: PLA_2 _venom genes in closely related *Sistrurus *rattlesnakes with divergent diets J Mol Evol 2008 66 151 166 10.1007/s00239-008-9067-7 18253686

[B61] van de Lagemaat LN Landry JR Mager DL Medstrand P Transposable elements in mammals promote regulatory variation and diversification of genes with specialized functions Trends Genet 2003 19 530 536 10.1016/j.tig.2003.08.004 14550626

[B62] Medstrand P van de Lagemaat LN Dunn CA Landry JR Svenback D Mager DL Impact of transposable elements on the evolution of mammalian gene regulation Cytogenet Genome Res 2005 110 342 345 10.1159/000084966 16093686

[B63] Zerbino DR Birney E Velvet: algorithms for *de novo *short read assembly using de Bruijn graphs Genome Res 2008 18 821 829 10.1101/gr.074492.107 18349386 PMC2336801

[B64] Kumar S Blaxter ML Comparing *de novo *assemblers for 454 transcriptome data BMC Genomics 2010 11 571 10.1186/1471-2164-11-571 20950480 PMC3091720

[B65] Flicek P Birney E Sense from sequence reads: methods for alignment and assembly Nature Methods 2009 6 S6 S12 10.1038/nmeth.1376 19844229

[B66] Liu Q Zhang X Yin W Li C Huang Y Qiu P Su X Hu S Yan G A catalog for transcripts in the venom gland of the *Agkistrodon acutus*: Identification of the toxins potentially involved in coagulopathy Biochem Biophys Res Commun 2006 341 522 531 10.1016/j.bbrc.2006.01.006 16438937

[B67] Naamati G Askenazi M Linial M ClanTox: a classifier of short animal toxins Nucleic Acid Res 2009 37 W363 W368 10.1093/nar/gkp299 19429697 PMC2703885

[B68] He Q He Q Deng X Yao L Meng E Liu Z Liang S ATDB: a uni-database platform for animal toxins Nucleic Acids Res 2008 36 D293 297 17933766 10.1093/nar/gkm832 PMC2238984

[B69] Rawlings ND Barrett AJ Bateman A MEROPS: the peptidase database Nucleic Acids Res 2010 38 D227 233 10.1093/nar/gkp971 19892822 PMC2808883

[B70] Fox JW Ma L Nelson K Sherman NE Serrano SMT Comparison of indirect and direct approaches using ion-trap and Fourier transform ion cyclotron resonance mass spectrometry for exploring viperid venom proteomes Toxicon 2006 47 700 714 10.1016/j.toxicon.2006.01.022 16574175

[B71] Georgieva D Öhler M Seifert J von Bergen M Arni RK Genov N Betzel C Snake venomic of *Crotalus durissus terrificus*. Correlation with pharmacological activities J Proteome Res 2010 9 2302 2316 10.1021/pr901042p 20205475

[B72] Calvete JJ Fasoli E Sanz L Boschetti E Righetti PG Exploring the venom proteome of the western diamondback rattlesnake, *Crotalus atrox*, via snake venomics and combinatorial peptide ligand library approaches J Proteome Res 2009 8 3055 3067 10.1021/pr900249q 19371136

[B73] Wermelinger LS Dutra DLS Oliveira-Carvalho AL Soares MR Bloch JrC Zingali RB Fast analysis of low molecular mass compounds present in snake venom: identification of ten new pyroglutamate-containing peptides Rapid Commun Mass Spectrom 2005 19 1703 1708 10.1002/rcm.1973 15912471

[B74] Menin L Perchuć A Favreau P Perret F Michalet S Schöni R Wilmer M Stöcklin R High throughput screening of bradykinin-potentiating peptides in *Bothrops moojeni *snake venom using precursor ion mass spectrometry Toxicon 2008 51 1288 1302 10.1016/j.toxicon.2008.02.019 18471845

[B75] Munekiyo SM Mackessy SP Presence of peptide inhibitors in rattlesnake venoms and their effects on endogenous metalloproteases Toxicon 2005 45 255 263 10.1016/j.toxicon.2004.10.009 15683863

[B76] Wagstaff SC Favreau P Cheneval O Laing GD Wilkinson MC Miller RL Stöckin R Harrison RA Molecular characterisation of endogenous snake venom metalloproteinase inhibitors Biochem Biophys Res Commun 2008 365 650 656 10.1016/j.bbrc.2007.11.027 18029259

[B77] Huang KF Chiou SH Ko TP Wang AH-J Determinants of the inhibition of a Taiwan habu venom metalloprotease by its endogenous inhibitors revealed by X-ray crystallography and synthetic inhibitor analogues Eur J Biochem 2002 269 3047 3056 12071970 10.1046/j.1432-1033.2002.02982.x

[B78] Castoe TA Poole AW Gu W Koning J Daza JM Smith EN Pollock DD Rapid identification of thousands of copperhead snake (*Agkistrodon contortrix*) microsatellite loci from modest amounts of 454 shotgun genome sequence Mol Ecol Res 2010 10 341 347 10.1111/j.1755-0998.2009.02750.x PMC5172459 21565030

[B79] Fry BG Roelants K Champagne DE Scheib H Tyndall JDA King GF Nevalainen TJ Norman JA Lewis RJ Norton RS Renjifo C Rodríguez de la Vega RC The toxicogenomic multiverse: convergent recruitment of proteins into animal venoms Annu Rev Genom Human Genet 2009 10 483 511 10.1146/annurev.genom.9.081307.164356 19640225

[B80] Nei M Gu X Sitnikova T Evolution by the birth-and-death process in multigene families of the vertebrate immune system Proc Natl Acad Sci USA 1997 94 7799 7806 10.1073/pnas.94.15.7799 9223266 PMC33709

[B81] Fry BG From genome to 'venome': molecular origin and evolution of the snake venom proteome inferred from phylogenetic analysis of toxin sequences and related body proteins Genome Res 2005 15 403 420 10.1101/gr.3228405 15741511 PMC551567

[B82] Kashima S Roberto PG Soares AM Astolfi-Filho S Pereira JO Giuliati S Faria JrM Xavier MAS Fontes MRM Giglio JR França SC Analysis of *Bothrops jararacussu *venomous gland transcriptome focusing on structural and functional aspects: gene expression profile of highly expressed phopholipases A_2_ Biochimie 2004 86 211 239 10.1016/j.biochi.2004.02.002 15134836

[B83] Cidade DAP Simão TA Dávila AMR Wagner G Junqueira-de-Azevedo ILM Ho PL Bon C Zingali RB Albano RM *Bothrops jararaca *venom gland transcriptome: analysis of the gene expression pattern Toxicon 2006 48 437 461 10.1016/j.toxicon.2006.07.008 16905169

[B84] Neiva M Arraes FB de Souza JV Rádis-Baptista G Prieto da Silva AR Walter ME Brigido MM Yamane T López-Lozano JL Astolfi-Filho S Transcriptome analysis of the Amazonian viper *Bothrops atrox *venom gland using expressed sequence tags (ESTs) Toxicon 2009 53 427 4 10.1016/j.toxicon.2009.01.006 19708221

[B85] Doley R Kini RM Protein complexes in snake venom Cell Mol Life Sci 2009 66 2851 2871 10.1007/s00018-009-0050-2 19495561 PMC11115964

[B86] Juárez P Comas I González-Candelas F Calvete JJ Evolution of snake venom disintegrins by positive Darwinian selection Mol Biol Evol 2008 25 2391 2407 10.1093/molbev/msn179 18701431

[B87] Lu Q Navdaev A Clemetson JM Clemetson KJ Snake venom C-type lectins interacting with platelet receptors. Structure-function relationships and effects on haemostasis Toxicon 2005 45 1089 1098 10.1016/j.toxicon.2005.02.022 15876445

[B88] Walker JR Nagar B Young NM Hirama Y Rini JM X-ray crystal structure of a galactose-specific C-type lectin possessing a novel decameric quaternary structure Biochemistry 2004 43 3783 3792 10.1021/bi035871a 15049685

[B89] Freedman RB Hirst TR Tuite MF Protein disulphide isomerase: building bridges in protein folding Trends Biochem Sci 1994 19 331 336 10.1016/0968-0004(94)90072-8 7940678

[B90] Yao Y Zhou Y Wang C Both the isomerase and chaperone activities of protein disulfide isomerase are required for the reactivation of reduced and denatured acidic phospholipase A_2_ EMBO J 1997 16 651 658 10.1093/emboj/16.3.651 9034346 PMC1169667

[B91] Fleischmann J Liu H Wu CP Polyadenylation of ribosomal RNA by *Candida albicans *also involves the small subunit BMC Mol Biol 2004 5 17 10.1186/1471-2199-5-17 15461824 PMC522811

[B92] Kuai L Fang F Butler JS Sherman F Polyadenylation of rRNA in *Saccharomyces cerevisiae* Proc Natl Acad Sci USA 2004 101 8581 8586 10.1073/pnas.0402888101 15173578 PMC423237

[B93] Decuypere S Vandesompele J Yardley V De Donckeri S Laurent T Rijal S Llanos-Cuentas A Chappuis F Arevalo J Dujardin JC Differential polyadenylation of ribosomal RNA during post-transcriptional processing in Leishmania Parasitology 2005 131 121 329 10.1017/S0031182005007341 16178353

[B94] Slomovic S Laufer D Geiger D Schuster G Polyadenylation and degradation of human mitochondrial RNA: the prokaryotic past leaves its mark Mol Cell Biol 2005 25 6427 6435 10.1128/MCB.25.15.6427-6435.2005 16024781 PMC1190340

[B95] Pahari S Mackessy SP Kini RM The venom gland transcriptome of the Desert Massasauga rattlesnake (*Sistrurus catenatus edwardsii*): towards an understanding of venom composition among advanced snakes (Superfamily Colubroidea) BMC Mol Biol 2007 8 115 10.1186/1471-2199-8-115 18096037 PMC2242803

[B96] Arava Y Wang Y Storey JD Liu CL Brown PO Herschlag D Genome-wide analysis of mRNA translation profiles in *Saccharomyces cerevisiae* Proc Natl Acad Sci USA 2003 100 3889 3894 10.1073/pnas.0635171100 12660367 PMC153018

[B97] Preiss T Baron-Benhamou J Ansorge W Hentze MW Homodirectional changes in transcriptome composition and mRNA translation induced by rapamycin and heat shock Nat Struct Mol Biol 2003 10 1039 1047 10.1038/nsb1015 14608375

[B98] MacKay VL Li X Flory MR Turcott E Law GL Serikawa KA Xu XL Lee H Goodlett DR Aebersold R Zhao LP Morris DR Gene expression in yeast responding to mating pheromone: Analysis by high-resolution translation state analysis and quantitative proteomics Mol Cell Proteomics 2004 3 478 489 10.1074/mcp.M300129-MCP200 14766929

[B99] Law GL Bickel KS MacKay VL Morris DR The undertranslated transcriptome reveals widespread translational silencing by alternative 5' transcript leaders Genome Biol 2005 6 R111 10.1186/gb-2005-6-13-r111 16420678 PMC1414110

[B100] Fernández J Lomonte B Sanz L Angulo Y Gutiérrez JM Calvete JJ Snake venomics of *Bothriechis nigroviridis *reveals extreme variability among palm pitviper venoms: different evolutionary solutions for the same trophic purpose J Proteome Res 2010 9 4234 4241 10.1021/pr100545d 20590130

[B101] Castoe TA Daza JM Smith EN Sasa MM Kuch U Campbell JA Chippindale PT Parkinson CL Comparative phylogeography of pitvipers suggests a consensus of ancient Middle American highland biogeography J Biogeogr 2009 36 88 103 10.1111/j.1365-2699.2008.01991.x

[B102] Crother BI Campbell JA Hillis DM Campbell JA, Brodie EDJr Phylogeny and historical biogeography of the palm pitvipers, genus Bothriechis: biochemical and morphological evidence Biology of the Pitvipers 1992 Selva: Tyler, TX 1 19

[B103] Kuhner MK Felsenstein J A simulation comparison of phylogeny algorithms under equal and unequal evolutionary rates Mol Biol Evol 1994 11 459 468 8015439 10.1093/oxfordjournals.molbev.a040126

[B104] Deng HW Fu YX The effects of variable mutation rates across sites on the phylogenetic estimation of effective population size or mutation rate of DNA sequences Genetics 1996 144 1271 1281 8913767 10.1093/genetics/144.3.1271 PMC1207618

[B105] Yang Z Among-site rate variation and its impact on phylogenetic analyses Tree 1996 11 367 372 21237881 10.1016/0169-5347(96)10041-0

[B106] Castoe TA Sasa MM Parkinson CL Modeling nucleotide evolution at the mesoscale: the phylogeny of the Neotropical pitvipers of the *Porthidium *group (Viperidae: Crotalinae) Mol Phylogenet Evol 2005 37 881 898 10.1016/j.ympev.2005.05.013 16024260

[B107] Castoe TA Parkinson CL Bayesian mixed models and the phylogeny of pitvipers (Viperidae: Serpentes) Mol Phylogenet Evol 2006 39 91 110 10.1016/j.ympev.2005.12.014 16504544

[B108] Castoe TA Chippindale PT Campbell JA Ammermann LK Parkinson CL Molecular systematics of the Middle American jumping pitvipers (genus Atropoides) and phylogeography of the *Atropoides nummifer *complex Herpetologica 2003 59 420 431 10.1655/01-105.2

[B109] Paine MJ Desmond HP Theakston RDG Crampton JM Gene expression in *Echis carinatus *(carpet viper) venom glands following milking Toxicon 1992 30 379 386 10.1016/0041-0101(92)90534-C 1626320

[B110] Margulies M Egholm M Altman WE Attiya S Bader JS Bemben LA Berka J Braverman MS Chen Y Chen Z Dewell SB Du L Fierro JM Gomes XV Godwin BC He W Helgesen S Ho CH Ho CH Irzyk GP Jando SC Alenquer MLI Jarvie TP Jirage KB Kim J Knight JR Lanza JR Leamon JH Lefkowitz SM Lei M Li J Lohman KL Lu H Makhijani VB McDade KE McKenna MP Myers EW Nickerson E Nobile JR Plant R Puc BP Ronan MT Roth GT Sarkis GJ Simons JF Simpson JW Srinivasan M Tartaro KR Tomasz A Vogt KA Volkmer GA Wang SH Wang Y Weiner MP Yu P Begley RF Rothberg JM Genome sequencing in microfabricated high-density picolitre reactors Nature 2005 437 376 380 16056220 10.1038/nature03959 PMC1464427

[B111] Smit AFA Hubley R Green P RepeatMasker (release of January 2009) http://repeatmasker.org

[B112] Altschul SF Gish W Miller W Myers EW Lipman DJ Basic local alignment search tool J Mol Biol 1990 215 403 410 2231712 10.1016/S0022-2836(05)80360-2

[B113] Conesa A Götz S García-Gómez JM Terol J Talón M Robles M Blast2GO: a universal tool for annotation visualization and analysis in functional genomics research Bioinformatics 2005 21 3674 3676 10.1093/bioinformatics/bti610 16081474

[B114] The Gene Ontology Consortium Gene ontology: tool for the unification of biology Nat Genet 2000 25 25 29 10.1038/75556 10802651 PMC3037419

[B115] Lomonte B Escolano J Fernández J Sanz L Angulo Y Gutiérrez JM Calvete JJ Snake venomics and antivenomics of the arboreal neotropical pitvipers *Bothriechis lateralis *and *Bothriechis schlegelii* J Proteome Res 2008 7 2445 2457 10.1021/pr8000139 18444672

[B116] Angulo Y Escolano J Lomonte B Gutiérrez JM Sanz L Calvete JJ Snake venomics of Central American pitvipers: clues for rationalizing the distinct envenomation profiles of *Atropoides nummifer *and *Atropoides picadoi* J Proteome Res 2008 7 708 719 10.1021/pr700610z 18161938

[B117] Alape-Girón A Sanz L Escolano J Flores-Díaz M Madrigal M Sasa M Calvete JJ Snake venomics of the lancehead pitviper *Bothrops asper*: geographic individual and ontogenetic variations J Proteome Res 2008 7 3556 3571 10.1021/pr800332p 18557640

[B118] Calvete JJ Sanz L Cid P de la Torre P Flores-Díaz M dos Santos MC Borges A Bremo A Angulo Y Lomonte B Alape-Girón A Gutiérrez JM Snake venomics of the Central American rattlesnake *Crotalus simus *and the South American *Crotalus durissus *complex points to neurotoxicity as an adaptive paedomorphic trend along *Crotalus *dispersal in South America J Proteome Res 2010 9 528 544 10.1021/pr9008749 19863078

[B119] Papadopoulos JS Agarwala R COBALT: constraint-based alignment tool for multiple protein sequences Bioinformatics 2007 23 1073 1079 10.1093/bioinformatics/btm076 17332019

[B120] Edgar RC MUSCLE: a multiple sequence alignment method with reduced time and space complexity BMC Bioinformatics 2004 5 113 10.1186/1471-2105-5-113 15318951 PMC517706

