# Supplementary figures and images for: Profiling the venom gland transcriptomes of Costa Rican snakes by 454 pyrosequencing
Source: BMC Genomics. 2011 May 23;12:259. doi: 10.1186/1471-2164-12-259 (PMC3128066; doi:10.1186/1471-2164-12-259)

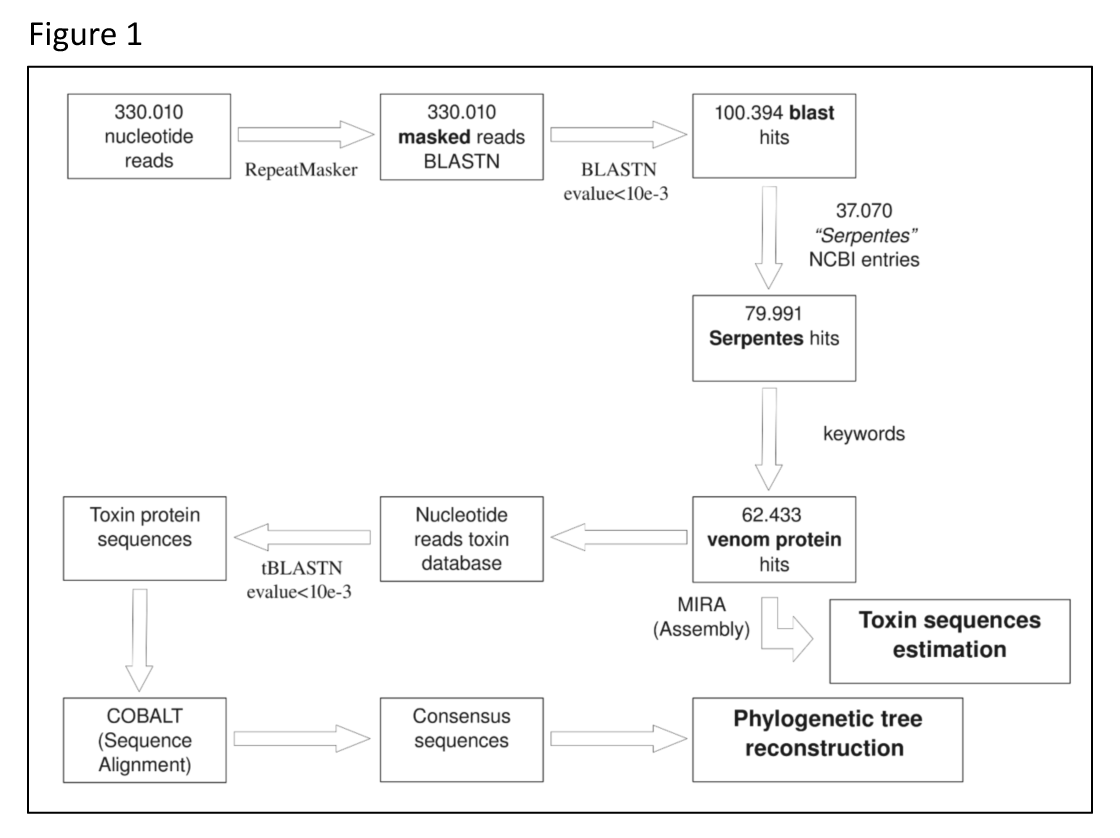

Supplement: Additional file 1 — Table S1: RepeatMasker usage results and features of the sequence elements masked in the 8 Costa Rican venom gland transcriptomes analyzed Table S2: Summary of the 454 sequencing statistics and annotation of transcripts in the 8 venom gland transcriptomes. Table S3: Number of reads aligned to translated (ORF) regions of reference snake venom toxin sequences. Table S4: Relative occurrence (in %) of the ORF-coding reads listed in Table S3. Table S5: Distribution of reads per contig among the SVMP genes. Table S6: Distribution of reads per contig among the PLA2 genes. Table S7: Distribution of reads per contig among the serine proteinase genes. [file 1471-2164-12-259-S1.TIFF]
