# Supplementary material for: Profiling the venom gland transcriptomes of Costa Rican snakes by 454 pyrosequencing
Source: BMC Genomics. 2011 May 23;12:259. doi: 10.1186/1471-2164-12-259 (PMC3128066; doi:10.1186/1471-2164-12-259)
Supplement: Additional file 2 — Figure S1. Summary of the strategy employed to assembly and analyze the 454 pyrosequencing reads from the venom gland transcriptomes of the Costa Rican snakes Bothrops asper (from Caribbean and Pacific populations), Bothriechis lateralis, Bothriechis schlegelii, Atropoides picadoi, Atropoides mexicanus, Crotalus simus, and Cerrophidion godmani. [file 1471-2164-12-259-S2.DOCX]

**Table S1**

RepeatMasker usage results and features of the sequence elements masked in the 8 Costa Rican venom gland transcriptomes analyzed.

number of length percentage
 elements occupied of sequence
 (nucleotides)

**Retroelements** 28609 3312010 5.30

SINEs: 816 85079 0.14
 Penelope 1263 137377 0.22
 LINEs: 22125 2709376 4.34
 CRE/SLACS 0 0 0.00
 L2/CR1/Rex 6322 761398 1.22
 R1/LOA/Jockey 2 170 0.00
 R2/R4/NeSL 295 34226 0.05
 RTE/Bov-B 13880 1742940 2.79
 L1/CIN4 363 33265 0.05
 LTR elements: 5668 517555 0.83
 BEL/Pao 0 0 0.00
 Ty1/Copia 19 2164 0.00
 Gypsy/DIRS1 1102 142726 0.23
 Retroviral 4461 363048 0.58

**DNA transposons** 2749 238354 0.38
 hobo-Activator 1390 96605 0.15
 Tc1-IS630-Pogo 630 97585 0.16
 En-Spm 59 3109 0.00
 MuDR-IS905 5 350 0.00
 PiggyBac 10 597 0.00
 Tourist/Harbinger 94 4845 0.01
 Other (Mirage, 0 0 0.00
 P-element, Transib)

**Rolling-circles** 0 0 0.00 **Unclassified** 1 69 0.00
**Total interspersed repeats** 3550433 5.68
**Small RNA** 57 3726 0.01
**Satellites** 379 23726 0.04
**Simple repeats** 13791 815188 1.31
**Low complexity** 16505 775609 1.24

**Table S2**

Summary of the 454 sequencing statistics and annotation of transcripts in the 8 venom gland transcriptomes.

| Snake species Total reads Mean length (nt) BLAST (% of total hits) *Serpentes* (% of total hits) Venom (% *Serpentes* hits)   hits hits protein |
| --- |
| *C. simus* 22389 193.6 3608 (16.1) 2320 (64.3) 1327 (57.2) |
| *B. asper* (Car) 123485 185.6 43818 (35.5) 35655 (81.4) 28220 (79.1) |
| *B. asper* (Pac) 16076 184.8 2848 (17.7) 2078 (72.9) 1378 (66.3) |
| *C. godmani* 44843 182.1 13743 (30.7) 11252 (81.9) 9824 (87.3) |
| *A. picadoi* 31027 188.8 13295 (42.8) 11350 (85.4) 9951 (87.7) |
| *A. mexicanus* 27080 192.1 6418 (23.7) 4070 (63.4) 3109 (76.4) |
| *B. schlegelii* 33276 191.5 6826 (20.5) 4525 (66.2) 2893 (63.8) |
| *B. lateralis* 31833 186.1 9838 (30.9) 7970 (81.0) 5731 (71.9) |
| **TOTAL** 330010 187.3 100394 (30.4) 79991 (79.7) 62433 (78.0) |

**Table S3**

Number of reads aligned to translated (ORF) regions of reference snake venom toxin sequences. Protein family names as in Table 1.

| ***C. simus*  *B. asper* (Car) *B. asper* (Pac)  *C. godmani*  *A. picadoi*  *A. mexicanus B. schlegelii*  *B. lateralis*** |
| --- |
|  |
| BPP 4 60 1 8 24 1 4 21 |
| CRISP 0 108 1 60 99 5 48 66 |
| CTL 9 115 12 8 127 2 0 43 |
| GF 4 72 4 3 19 1 4 18 |
| LAO 17 267 8 108 80 59 15 44 |
| PLA_2_ 29 1610 43 196 32 48 8 10 |
| SVMP 51 6746 276 1635 2562 649 458 1457 |
| SP 47 295 6 198 43 30 50 67 |
| 5'-NTase 1 112 2 38 10 18 1 23 |
| PDE 3 55 3 13 7 0 2 33 |
| GC 2 37 2 12 6 5 0 1 |
| WAP 0 6 0 0 0 0 0 0 |
| KUN 0 0 0 0 0 0 4 0 |
| KAZ 0 0 0 0 0 0 9 12 |
| HYA 2 7 0 3 1 1 0 2 |
| OHA 0 0 0 0 2 1 0 0 |
| 3FTx 1 0 0 0 0 0 0 0 |

**Table S4**

Relative occurrence (in %) of the ORF-coding reads listed in Table S3. The relative abundance (%) of proteins of the different families found in the venom proteomes^49-52^ are given in parentheses. The proteome of *C. godmani* venom has not been reported.

| ***C. simus* *B. asper* (Car) *B. asper* (Pac)  *C. godmani*  *A. picadoi*  *A. mexicanus B. schlegelii*  *B. lateralis*** |
| --- |
|  |
| BPP 2.3 (3.5) 0.6 (-) 0.3 (-) 0.4 0.8 (1.8) 0.1 (8.6) 0.7 (13.4) 1.2 (11.1) |
| CRISP 0 (-) 1.1 (0.1) 0.3 (0.1) 2.6 3.3 (1.9) 0.6 (1.9) 8.1 (2.1) 3.7 (6.5) |
| CTL 5.3 (-) 1.2 (0.5) 3.4 (0.5) 0.4 4.2 (1.3) 0.2 (1.3) 0 (-) 2.4 (0.9) |
| GF 2.3 (-) 0.5 (-) 0 (-) 0 0.3 (< 0.1) 0 (< 0.1) 0.7 (-) 0.05 (0.5) |
| LAO 10.0 (3.8) 2.8 (9.2) 2.3 (4.6) 4.7 2.7 (2.2) 7.2 (9.1) 2.5 (8.9) 2.5 (6.1) |
| PLA_2_ 17.1 (7.6) 17.0 (28.8) 12.1 (45.1) 8.6 1.1 (9.5) 5.9 (36.5) 1.3 (43.8) 0.5 (8.7) |
| SVMP 30.1 (71.7) 71.2 (41.0) 77.9 (44.0) 71.7 85.3 (66.4) 79.2 (17.2) 77.1 (17.7) 82.4 (55.1) |
| SP 27.6 (5.3) 3.1 (18.2) 1.7 (4.4) 8.7 1.4 (13.5) 3.7 (22) 8.4 (5.8) 3.8 (11.3) |
| 5'-NTase 0.6 (-) 1.2 (-) 0.6 (-) 1.7 0.3 (-) 2.2 (-) 0.1 (-) 1.3 (-) |
| PDE 1.8 (-) 0.6 (-) 0.8 (-) 0.6 0.2 (-) 0 (-) 0.2 (-) 1.8 (-) |
| GC 1.2 (-) 0.4 (-) 0.6 (-) 0.6 0.2 (-) 0.6 (-) 0 (-) 0.05 (-) |
| WAP 0 (-) 0.06 (-) 0 (-) 0 0 (-) 0 (-) 0 (-) 0 (-) |
| KUN 0 (-) 0 (-) 0 (-) 0 0 (-) 0 (-) 0.7 (-) 0 (-) |
| KAZ 0 (-) 0 (-) 0 (-) 0 0 (-) 0 (-) 1.5 (8.3) 0.6 (-) |
| HYA 1.2 (-) 0.06 (-) 0 (-) 1.3 0.03 (-) 0.1 (-) 0 (-) 0.1 (-) |
| OHA 0 (-) 0 (-) 0 (-) 0 0.06 (-) 0.1 (-) 0 (-) 0 (-) |
| 3FTx 0.6 (-) 0 (-) 0 (-) 0 0 (-) 0 (< 0.1) 0 (-) 0 (-) |

**Table S5**

Distribution of reads per contig among the SVMP genes listed in Table 3

| \| ***C. simus*** \| ***B. asper* (Car)** \| ***B. asper* (Pac)** \| ***C. godmani*** \| ***A. picadoi*** \| ***A. mexicanus*** \| ***B. schlegelii*** \| ***B. lateralis*** \| \| --- \| --- \| --- \| --- \| --- \| --- \| --- \| --- \| \|  \|  \|  \|  \|  \|  \|  \|  \| \| 7 \| 3590 \| 218 \| 719 \| 1051 \| 583 \| 94 \| 701 \| \| 5 \| 449 \| 9 \| 233 \| 507 \| 33 \| 65 \| 477 \| \| 5 \| 438 \| 6 \| 156 \| 412 \| 4 \| 54 \| 22 \| \| 4 \| 418 \| 5 \| 74 \| 152 \| 4 \| 47 \| 22 \| \| 4 \| 233 \| 4 \| 50 \| 72 \|  \| 28 \| 16 \| \| 4 \| 180 \|  \| 46 \| 54 \|  \| 25 \| 13 \| \| 4 \| 173 \|  \| 42 \| 21 \|  \| 21 \| 11 \| \| 3 \| 95 \|  \| 26 \| 20 \|  \| 16 \| 11 \| \| 3 \| 63 \|  \| 26 \| 14 \|  \| 16 \| 10 \| \|  \| 58 \|  \| 21 \| 13 \|  \| 13 \| 8 \| \|  \| 48 \|  \| 19 \| 11 \|  \| 10 \| 7 \| \|  \| 46 \|  \| 17 \| 11 \|  \| 9 \| 6 \| \|  \| 44 \|  \| 14 \| 9 \|  \| 7 \| 6 \| \|  \| 43 \|  \| 14 \| 8 \|  \| 7 \| 5 \| \|  \| 42 \|  \| 12 \| 8 \|  \|  \| 5 \| \|  \| 41 \|  \| 9 \|  \|  \|  \| 5 \| \|  \| 38 \|  \| 9 \|  \|  \|  \| 4 \| \|  \| 36 \|  \| 9 \|  \|  \|  \| 4 \| \|  \| 32 \|  \| 8 \|  \|  \|  \| 4 \| \|  \| 31 \|  \|  \|  \|  \|  \| 4 \| \|  \| 29 \|  \|  \|  \|  \|  \|  \| \|  \| 19 \|  \|  \|  \|  \|  \|  \| \|  \| 15 \|  \|  \|  \|  \|  \|  \| \|  \| 15 \|  \|  \|  \|  \|  \|  \| \|  \| 14 \|  \|  \|  \|  \|  \|  \| \|  \| 14 \|  \|  \|  \|  \|  \|  \| \|  \| 13 \|  \|  \|  \|  \|  \|  \| \|  \| 13 \|  \|  \|  \|  \|  \|  \| \| 12 \| \| \| \| \| \| \| \| |
| --- | --- | --- | --- | --- | --- | --- | --- | --- | --- | --- | --- | --- | --- | --- | --- | --- | --- | --- | --- | --- | --- | --- | --- | --- | --- | --- | --- | --- | --- | --- | --- | --- | --- | --- | --- | --- | --- | --- | --- | --- | --- | --- | --- | --- | --- | --- | --- | --- | --- | --- | --- | --- | --- | --- | --- | --- | --- | --- | --- | --- | --- | --- | --- | --- | --- | --- | --- | --- | --- | --- | --- | --- | --- | --- | --- | --- | --- | --- | --- | --- | --- | --- | --- | --- | --- | --- | --- | --- | --- | --- | --- | --- | --- | --- | --- | --- | --- | --- | --- | --- | --- | --- | --- | --- | --- | --- | --- | --- | --- | --- | --- | --- | --- | --- | --- | --- | --- | --- | --- | --- | --- | --- | --- | --- | --- | --- | --- | --- | --- | --- | --- | --- | --- | --- | --- | --- | --- | --- | --- | --- | --- | --- | --- | --- | --- | --- | --- | --- | --- | --- | --- | --- | --- | --- | --- | --- | --- | --- | --- | --- | --- | --- | --- | --- | --- | --- | --- | --- | --- | --- | --- | --- | --- | --- | --- | --- | --- | --- | --- | --- | --- | --- | --- | --- | --- | --- | --- | --- | --- | --- | --- | --- | --- | --- | --- | --- | --- | --- | --- | --- | --- | --- | --- | --- | --- | --- | --- | --- | --- | --- | --- | --- | --- | --- | --- | --- | --- | --- | --- | --- | --- | --- | --- | --- | --- | --- | --- | --- | --- | --- | --- | --- | --- | --- | --- | --- | --- | --- | --- | --- | --- | --- | --- | --- | --- | --- | --- | --- |

**Table S6**

Distribution of reads per contig among the PLA_2_ genes listed in Table 3

| \| ***C. simus*** \| ***B. asper* (Car)** \| ***B. asper* (Pac)** \| ***C. godmani*** \| ***A. picadoi*** \| ***A. mexicanus*** \| ***B. schlegelii*** \| ***B. lateralis*** \| \| --- \| --- \| --- \| --- \| --- \| --- \| --- \| --- \| \|  \|  \|  \|  \|  \|  \|  \|  \| \| 17 \| 529 \| 21 \| 103 \| 19 \| 41 \| 6 \| 4 \| \| 8 \| 395 \| 9 \| 48 \| 8 \| 3 \|  \| 3 \| \| 2 \| 266 \| 6 \| 28 \|  \|  \|  \| 3 \| \|  \| 159 \| 3 \| 2 \|  \|  \|  \|  \| \|  \| 59 \|  \|  \|  \|  \|  \|  \| \|  \| 42 \|  \|  \|  \|  \|  \|  \| \|  \| 18 \|  \|  \|  \|  \|  \|  \| \|  \| 18 \|  \|  \|  \|  \|  \|  \| \|  \| 15 \|  \|  \|  \|  \|  \|  \| |
| --- | --- | --- | --- | --- | --- | --- | --- | --- | --- | --- | --- | --- | --- | --- | --- | --- | --- | --- | --- | --- | --- | --- | --- | --- | --- | --- | --- | --- | --- | --- | --- | --- | --- | --- | --- | --- | --- | --- | --- | --- | --- | --- | --- | --- | --- | --- | --- | --- | --- | --- | --- | --- | --- | --- | --- | --- | --- | --- | --- | --- | --- | --- | --- | --- | --- | --- | --- | --- | --- | --- | --- | --- | --- | --- | --- | --- | --- | --- | --- | --- | --- | --- | --- | --- | --- | --- | --- | --- |

**Table S7**

Distribution of reads per contig among the serine proteinase (SP) genes listed in Table 3.

| \| ***C. simus*** \| ***B. asper* (Car)** \| ***B. asper* (Pac)** \| ***C. godmani*** \| ***A. picadoi*** \| ***A. mexicanus*** \| ***B. schlegelii*** \| ***B. lateralis*** \| \| --- \| --- \| --- \| --- \| --- \| --- \| --- \| --- \| \|  \|  \|  \|  \|  \|  \|  \|  \| \| 15 \| 48 \| 5 \| 122 \| 10 \| 9 \| 13 \| 10 \| \| 7 \| 38 \|  \| 7 \| 6 \| 6 \| 7 \| 10 \| \| 5 \| 37 \|  \| 7 \| 5 \| 5 \| 6 \| 7 \| \| 4 \| 21 \|  \| 7 \| 4 \| 4 \| 6 \| 6 \| \| 2 \| 21 \|  \| 6 \| 2 \| 2 \| 3 \| 4 \| \| 2 \| 16 \|  \| 3 \| 2 \| 2 \| 3 \| 4 \| \|  \| 14 \|  \| 3 \| 2 \|  \| 2 \| 4 \| \|  \| 12 \|  \| 3 \|  \|  \| 2 \| 4 \| \|  \| 11 \|  \| 3 \|  \|  \|  \| 3 \| \|  \| 10 \|  \| 3 \|  \|  \|  \| 3 \| \|  \| 9 \|  \| 2 \|  \|  \|  \| 2 \| \|  \| 9 \|  \| 2 \|  \|  \|  \|  \| \|  \| 9 \|  \| 2 \|  \|  \|  \|  \| \|  \| 7 \|  \|  \|  \|  \|  \|  \| \|  \| 7 \|  \|  \|  \|  \|  \|  \| |
| --- | --- | --- | --- | --- | --- | --- | --- | --- | --- | --- | --- | --- | --- | --- | --- | --- | --- | --- | --- | --- | --- | --- | --- | --- | --- | --- | --- | --- | --- | --- | --- | --- | --- | --- | --- | --- | --- | --- | --- | --- | --- | --- | --- | --- | --- | --- | --- | --- | --- | --- | --- | --- | --- | --- | --- | --- | --- | --- | --- | --- | --- | --- | --- | --- | --- | --- | --- | --- | --- | --- | --- | --- | --- | --- | --- | --- | --- | --- | --- | --- | --- | --- | --- | --- | --- | --- | --- | --- | --- | --- | --- | --- | --- | --- | --- | --- | --- | --- | --- | --- | --- | --- | --- | --- | --- | --- | --- | --- | --- | --- | --- | --- | --- | --- | --- | --- | --- | --- | --- | --- | --- | --- | --- | --- | --- | --- | --- | --- | --- | --- | --- | --- | --- | --- | --- | --- |
